# Genetic characteristics of the diploid offsprings in potato Cooperation 88 induced by diploid donor IVP101

**DOI:** 10.3389/fpls.2024.1486549

**Published:** 2024-11-08

**Authors:** Rongyan Wang, Yan Feng, Jing Peng, Chen Tan, Jian Zhou, Yang Hai, Youwei Luo, Dahai Hao, Canhui Li, Wei Tang

**Affiliations:** ^1^ Yunnan Key Laboratory of Potato Biology, Yunnan Normal University, Kunming, China; ^2^ School of Life Sciences, Yunnan Normal University, Kunming, China; ^3^ School of Economics, Yunnan Normal University, Kunming, China; ^4^ Yunnan YinMore Modern Agriculture Co., Ltd., Kunming, China; ^5^ Dehong Agricultural Technology Extension Center, Mangshi, China

**Keywords:** potato diploid breeding, distant hybridization, genomic elimination, chloroplast genome, inheritance patterns, comparative analysis

## Abstract

Diploid lines (2n = 2x = 24) derived from tetraploid potato cultivars have been utilized to hybridize with wild diploid potato species, yielding fertile offsprings. Utilizing the pollen of *Solanum tuberosum* Group Phureja, such as IVP101, IVP35 and IVP48, as an inducer for wide hybridization with tetraploid cultivars represents a common method for producing diploids. In this study, we created a distant hybridization induced population of tetraploid potato cultivar Cooperation 88 (C88) and IVP101, and screened all diploids using flow cytometry and ploidyNGS. We investigated the genetic composition of chloroplast and nuclear genomes in 43 diploid offsprings. We found that all diploid offsprings share the same chloroplast genomic sequence as C88 and no evidence of paternal chloroplast inheritance was found. Used SNP data to calculate the theoretical introgression index of IVP101 with diploid offsprings. The results showed that the inducer’s nuclear genome was involved in the nuclear genome of the diploid offsprings with purple stem trait, indicating that the inducer nuclear genome was not completely eliminated in the nuclear genome during distant hybridization. Furthermore, we conducted a comparative analysis of the chloroplast genomes of the *Solanum* genus. The results indicated that (1) the chloroplast genome sizes of the 14 *Solanum* species ranged from 154,289 bp to 155,614 bp, with a total number of genes ranging 128-141, and with *ycf*1 and *rps*19 pseudogenes appearing at the IRB/SSC and IRA/LSC boundaries, respectively; (2) eight divergent hotspots distributed in the LSC and SSC regions of the *Solanum* chloroplast genomes were identified; (3) positive selection was detected in the *clp*P, *rbc*L, *rps*15, and *rps*4 genes, likely contributing to the adaptation of Solanum species to different habitats. These results reveal the variation and evolutionary characteristics of chloroplast genomes in *Solanum* plants.

## 1 Introduction

Potato (*Solanum tuberosum* L.) is a species of the *Solanum* genus originating from the Andes Mountains in South America (Devaux et al., 2014). It is the world’s fourth largest cereal crop and the most important non-grain cereal crop (Devaux et al., 2014). Research and production of potatoes have traditionally focused on tetraploid cultivated potatoes, which have a complex genetic background and pose challenges to understand, thereby slowing down genetic improvement processes (Watanabe, 2015). Currently, the field of potato research is shifting towards using diploids instead of tetraploids for breeding. Techniques such as haploid induction (Zhang et al., 2022), distant hybridization induction (Cox et al., 2018), and *in vitro* culture of anthers, pollen, or ovary tissues are employed to create diploid potato materials (Du et al., 2021). Among these techniques, distant hybridization induction has been widely used in plants and lower organisms. Notable inducers utilized in the research of diploid potato induction through distant hybridization include IVP35, IVP48, and IVP101 (Amundson et al., 2020). The mechanism of potato diploidy induced by distant hybridization remains unclear, with three possible mechanisms identified. The first possible mechanism is gynogenesis, with the resulting diploids are referred to as dihaploids (Stanley et al., 1996). The second possibility involves chromosome recombination of the two parents, followed by complete or partial elimination of the inducer genome from the embryo after successful fertilization (Amundson et al., 2020). The third potential mechanism is chromosome recombination of the two parents, with no elimination of the inducer genome, leading to reduction of the recombined chromosomes (Ye et al., 2018). The latter two distant hybridization-induced chromosomal reduction mechanisms indicate the possible occurrence of inducer genome infiltration in the offspring, implying potential trait segregation. Recent reports using SNP genotyping (Bartkiewicz et al., 2018) and whole-genome sequencing technologies (Pham et al., 2019) have identified DNA segments (> 100 bp) from the inducer in the nuclear genome of potato distant hybridization offspring. Using polymorphic marker technology, Amundson et al. found that the nuclear genomes of eight dihaploids retained genomes from the inducer, six of which had intact chromosomes and two had large genomic segments (Amundson et al., 2021). However, the role of potato inducer in cytoplasmic inheritance in offspring is less reported in the literature.

Plant cells contain multiple chloroplast genomes and mitochondrial genomes that differ from nuclear genome inheritance. The chloroplast DNA (cpDNA) and the mitochondrial DNA (mtDNA) of the potato together constitute the potato cytoplasmic type. Hosaka and Sanetomo developed five markers for rapid identification of cytoplasmic types in potatoes, including four chloroplast genomic markers and one mitochondrial genomic marker (Hosaka and Sanetomo, 2012). This classification system divides potato cytoplasm into six types: M (Mother), A (Andigena), P (Phureja), W (Wild), D (Demissum), and T (Tuberosum), which has been validated in cultivated potatoes and their wild relatives (Hosaka and Sanetomo, 2012). The small size of the chloroplast genome, its limited gene count, low recombination frequency, stable linkage disequilibrium between different loci, and high conservation make the chloroplast genome play significant roles in species identification, parentage verification, molecular marker-assisted selection, gene editing, and genetic engineering (Daniell et al., 2016; Wambugu et al., 2015; Ortega and Lopez-vizcon, 2012; Apel and Bock, 2009). The chloroplast genome exhibits various configurations, including circular double-stranded, D-loop, linear, and net-like configurations, with the circular double-stranded being the most common configuration in angiosperms (Dobrogojski et al., 2020). The chloroplast genome of angiosperms displays a highly conserved tetrad structure with inverted repeat regions A (IRA) and B (IRB) of opposite directions but equal lengths, separated by a long single copy region (LSC) and a short single copy region (SSC) (Yurina et al., 2017). Chloroplast genome sizes from different plant taxa typically range from 100 to 200 kb, while most angiosperm chloroplast genomes range from 120 to 170 kb (Dobrogojski et al., 2020). The chloroplast genomes of most plants exhibit highly conserved features in terms of gene number and gene arrangement, usually comprising 120 to 150 genes, including around 80 protein-coding genes, 4 rRNA genes, and about 30 tRNA genes, which perform various functions in plant physiological processes (Mehmood et al., 2019).

The vast majority of angiosperms have maternal origins for their chloroplast genomes, while the chloroplast genome of gymnosperms originates from the male parent, for example, chloroplast transmission in Pinaceae is thought to be exclusively patrilineal (Zhang et al., 2003). However, Ni et al. found that the chloroplast genome of *Pinus massoniana* in distant hybrid offsprings exhibited a high frequency of paternal inheritance (more than 95%) and a low frequency of maternal inheritance by studying the chloroplast genome transmission (Ni et al., 2021). Additionally, biparental inheritance patterns of chloroplast genomes have been observed multiple times in angiosperms, particularly in plant groups with cytonuclear incompatibility (Jansen and Ruhlman, 2012). With a deeper investigation into chloroplast inheritance, researchers have noted potential evolutionary changes in the chloroplast genome inheritance patterns of angiosperms (Chung et al., 2023). Specifically, when maternal transmission is not strict in plants, there may be introgression of paternal chloroplast genomes, as seen in species like *Nicotiana tabacum* (Ruf et al., 2007) and *Arabidopsis thaliana* (Azhagiri and Maliga, 2007), where the chloroplast genome is maternally inherited but instances of pollen-mediated chloroplast genomes transfer to offspring have also been documented.

Potato variety Cooperation 88(C88) is a high-yielding tetraploid with excellent tuber quality, showing good adaptability to different environmental conditions and strong resistance to Potato late blight (Li et al., 2011). With a planting area exceeding 400,000 hectares in Yunnan Province, accounting for two-thirds of the province’s total potato planting area, this variety has become one of the main cultivated potato varieties in the region (Myrick et al., 2021). Therefore, utilizing C88 as the female parent for breeding materials shows promising prospects. As a pollinator of *S. phureja* origin, IVP101 is homozygous, with embryonic spots in the seeds and a large amount of purple pigment deposition on the stems (Straadt and Rasmussen, 2003). Compared with IVP35 and IVP48, it has a higher diploid induction rate (Straadt and Rasmussen, 2003). It holds greater potential for practical applications and serves as the primary inducer. In some potato diploid inducing systems, stem color and embryo spot is often used to distinguish between possible diploid (with maternal stem color and without spots) seeds and hybrid seeds (with paternal stem color and with spots). Researchers have found two dominant genes that control the pigment deposition in the plants, namely the *P* gene and the *R* gene. The expression of either gene can promote the deposition of purple pigment in the stems of seedlings (Dodds and Long, 1955; Hermsen and Verdenius, 1973). In addition, Dodds and Long found that the combination of dominant genes *B^c^* and *B^d^* with *P* or *R* at the cotyledon nodes of embryos can control the pigment deposition traits of cotyledon nodes. When the genotype is *P*_*B^c^* _ or *P*_*B^d^* _, blue spots are displayed; When the genotype is *pp B^d^* _*R*, red spots are displayed; When the genotype is *pp B^c^* _*R*, *pp B^c^* _*RR*, or *pp B^d^* _*RR*, there are no spots (Dodds and Long, 1956). C88 is an Andean subspecies with W/α[D] cytoplasmic type, while IVP101 is a P-type cytoplasmic type (Mori et al., 2012). There are few studies on whether the diploid offspring produced by distant hybridization will show polymorphic cytoplasmic types and whether the chloroplast genome inheritance pattern of the diploid offspring will change. Therefore, this study utilized C88 as the maternal material, IVP101 as the inducer for creating the distant hybridization induced population, to screen for diploid lines, identify the cytoplasmic types of diploid offsprings, determine the inheritance mode of the chloroplast genome in diploid offsprings, and preliminarily speculate on the potential mechanism of the induction of diploid offsprings by IVP101. Additionally, comparative analysis of the chloroplast genomes of C88, IVP101, and other *Solanum* species was conducted to reveal sequence variations and evolutionary characteristics of *Solanum* chloroplast genomes.

## 2 Materials and methods

### 2.1 Distant hybridization induced population construction

Harvest the pollinator IVP101 flowers at their peak, swiftly bring them back to the laboratory, and extract pollen grains using a dissecting needle. Assess pollen viability using the TTC (2,3,5-triphenyltetrazolium chloride, TTC) staining method. Before C88 flowering, remove its stamens with sterilized forceps. Dip a brush in IVP101 pollen with a viability rate exceeding 60% and brush it onto the stigma of each C88 flower, ensuring that yellow pollen is visible on the stigma. After pollination, bag the flowers with a 500-mesh nylon net to prevent interference from insects or extraneous pollen. Upon fruit ripening, collect the fruit, extract the seeds, air dry them, and screen for seeds with dominant purple embryo spots to indicate IVP101 hybridization (Hermsen and Verdenius, 1973). Sow the plump seeds without embryonic spots in a culture tube containing basic medium (3% sucrose, pH 5.7) (Murashige and Skoog, 1962).

### 2.2 Extraction of DNA and genome sequencing

Take fresh leaves from C88, IVP101, and the distant hybrid-induced population. Extract total DNA from the leaves using a plant DNA extraction kit (Tiangen Biotech Co., Ltd, China) and perform quality evaluation of the extracted DNA using 0.8% agarose gel electrophoresis. After passing the quality assessment, send the samples to BGI Genomics Beijing for second- and third-generation whole-genome resequencing, with sequencing depth of Illumina Novoseq 30× (PE150) + ONT 10×.

### 2.3 Ploidy analysis

#### 2.3.1 Ploidy analysis by flow cytometry

Fresh leaves of C88, IVP101, and their offspring were collected, rinsed with ddH_2_O, air-dried, and placed in 6 cm petri dishes according to their respective numbers. Subsequently, 1 mL of pre-chilled lysis buffer (prepared by mixing 4.574 g of magnesium chloride, 4.411 g of sodium citrate, 2.093 g of Mops, and 0.5 mL of TritonX-100 in 500 mL) was added to the petri dishes. Using a sterilized blade, the leaves were chopped in a consistent direction, followed by the addition of another 1 mL of pre-chilled lysis buffer. The samples were then refrigerated at 4°C for 30 minutes. Leaf debris was filtered out using a 400-mesh nylon filter, and the filtrate was transferred to 1.5 mL centrifuge tubes and kept in the dark at 4°C for 10 minutes. The tubes were centrifuged for 5 minutes at 1000 rpm in a low-temperature centrifuge, the supernatant was discarded, and 300 μL of pre-chilled 1×Propidium Iodide staining solution was added to the pellet. After gentle mixing, the samples were left in the dark at 4°C for 20 minutes before being subjected to further analysis.

#### 2.3.2 Ploidy analysis by ploidyNGS

Using CLC Genomics Workbench v.20.0.3 software for genome analysis, the sequenced clean reads were mapped to the No.9 chromosome of the reference genome DM 8.1, yielding mapped BAM file. Ploidy were assessment by ploidyNGS following the parameter: inputting the BAM file (–bam) and the string for generating the output file (–out), adjusting the –max_allele_freq parameter to 0.9 based on the interpretation guidelines for different ploidy levels published on the official website of ploidyNGS (https://github.com/diriano/ploidyNGS), the specific ploidy level determinations are as follows: (1) if the x-coordinate corresponding to the highest peak of Allele Freq is 50 and only the count positions of this peak exceed 100,000, it is classified as diploid; (2) if, besides the x-coordinates at 0 and 50, only the x-coordinates at 33.33 and 66.67 correspond to count positions reaching 100,000, it is identified as triploid; (3) if only the x-coordinates at 50, 33.33, and 66.67 correspond to count positions reaching 100,000, it is classified as tetraploid. (4) All other cases are considered polyploid (Augusto Corrêa Dos Santos et al., 2017).

### 2.4 Identification of cytoplasmic genomic polymorphisms

Using specific primers strict to the potato cytoplasmic genome (**Table 1**), DNA from C88, IVP101, and their diploid offsprings was amplified, followed by PCR and enzyme digestion. Band sizes were detected using 1.5% and 4% agarose gel electrophoresis. The control variety was Atlantic (T-type cytoplasm), ddH_2_O as template for blank control. PCR reaction system consists of 10 μL 2× Taq PCR Master Mix, 0.5 μL of upstream primer (10 μM), 0.5 μL of downstream primer (10 μM), 1 μL DNA template (concentration 50 ng·μL^-1^), and 8 μL ddH_2_O. PCR reaction procedure is as follows: Pre-denaturation at 95°C for 10 min, denaturation at 94°C for 30 s, annealing for 30 s (annealing temperature for each primer in **Table 1**), extension at 72°C for 1 min, 30 cycles; final extension at 72°C for 5 min.

**Table 1:** Primers for cytoplasmic genome labelling in potato.

Primer name	Primer sequence (5ˊ-3ˊ)	Expansion site	Annealing temperature/°C	Band size/bp
T	F: GGAGGGGTTTTTCTTGGTTG R: AAGTTTACTCACGGCAATCG	*trn*V-UAC/*ndh*C	55	446
				405
				205
S	F: GGTTCGAATCCTTCCGTC R: GATTCTTTCGCATCTCGATTC	*rps*16/*trn*Q	60	150
SAC	F: TTGGAGTTGTTGCGAATGAG R: GTTCCCTAGCCACGATTCTG	*1*/*11a*	60	150
A	F: AACTTTTTGAACTCTATTCCTT AATTG R: ACGCTTCATTAGCCCATACC	*10*	60	250
D	F: CGGGAGGTGGTGTACTTTCT R: ACGGCTGACTGTGTGTTTGA	*Band* 1	60	527

T, S, SAC, and A primers are chloroplast genome labeling primers, and D primers are mitochondrial genome labeling primers (Hosaka and Sanetomo, 2012).

Enzyme digestion reaction system and procedure: 1 μL 10× Quick Cut Buffer, 1 μL *Bam*H I enzyme (2U), 5 μL PCR product, 3 μL ddH_2_O, mix well, and incubate at 30°C for 30 min.

### 2.5 Chloroplast genome assembly and annotation

Utilize CLC Genomics Workbench v.20.0.3 to align sequencing data with the reference genome (NC_008096.1). Then, assemble the chloroplast genome using SPAdes v.3.15.4, setting the -phred-offset to 33 while maintaining default values for other options. After assembly, visualize the assembled FASTG file with Bandage. Manually resolve the “dumbbell” structure and remove overlapping ends to obtain a complete chloroplast genome. Align the raw sequencing data to the complete chloroplast genome using Bowtie2 (version 2.3.5.1, –very-sensitive-local). Perform a manual check on coverage to ensure accurate assembly. Use CPGAVAS2 (http://47.96.249.172:16019/analyzer/annotate) for chloroplast genome annotation and manually correct the annotation results. Utilize CPGview v.0.07 to examine and extract annotation information. This will illustrate the cis-splicing and trans-splicing genes within the chloroplast genome and generate a physical map of the chloroplast genome (Liu et al., 2023). Finally, submit the results to NCBI to obtain accession numbers. The accession numbers representing the IVP101 and C88 chloroplast genomes are PP680311 and PP680310, respectively.

### 2.6 Chloroplast genomic variant site detection

Using the PP680310 as the reference genome, CLC Genomics Workbench v.20.0.3 was used to detect genetic variation sites in the PP680311 and its diploid offsprings. By comparing shared genetic variation sites with each 5 bp as a step between IVP101 and the diploid offspring, to determine if the introgression has occurred during distant hybridization (if neighboring introgression sites are less than 5 bp, they are recorded as the same introgression site).

### 2.7 Nuclear genome introgression analysis

Using the diploid potato variety DM v8.1 as a reference, we analyzed SNP variations in IVP101, the diploid offspring, and the nuclear genome of *S. lycopersicum* (SRR1480889) using CLC Genomics Workbench v.20.0.3. We filtered SNPs with Plink v.1.9, retaining only biallelic SNPs (Malinsky et al., 2021). We employed the Dtrios module in Dsuite to calculate theoretical introgression values and assigned samples to groups (https://github.com/millanek/Dsuite): H1 for the green-stem diploid offsprings, H2 for the purple-stem diploid offsprings, and H3 for IVP101, with *S. lycopersicum* serving as the outgroup. Using TreeMix v.1.13, we infer the direction of introgression among populations again, setting the root to *S. lycopersicum*. We used m values ranging from 1 to 10, with each m value repeated three times (Pickrell and Pritchard, 2012).

### 2.8 Comparative analysis of chloroplast genomes in the genus *Solanum*

#### 2.8.1 Basic characteristics of the chloroplast genome in the genus *Solanum*

After acquiring the complete chloroplast genome sequence, we utilized CLC Genomics Workbench v.20.0.3 to conduct a preliminary comparison of genomic features, including total sequence length, lengths of four regions, overall GC content, and gene content. The chloroplast genome data of the other 12 *Solanum* species were obtained from the NCBI database, and detailed information was listed in **Supplementary Table 1**.

#### 2.8.2 SC/IR boundary

The IR region of chloroplast genomes in plants is regarded as the most conserved area. However, the boundary sequences may either expand outward or contract inward. This dynamic leads to variations in the copy number of associated genes and can result in the emergence of pseudogenes in the boundary regions (Yang et al., 2023). These phenomena represent common occurrences in chloroplast genome evolution and serve as primary contributors to length variation in the genomes. To investigate the contraction and expansion characteristics of the IR boundaries in the genus *Solanum*, we utilized IRscope (https://irscope.shinyapps.io/irapp/) to obtain and compare the boundary genes of IRA/IRB, LSC, and SSC in *Solanum* chloroplast genomes, as well as to assess their sequence lengths and distances from the boundaries.

#### 2.8.3 Mutation hotspots and gene analysis

Nucleotide polymorphism (Pi) serves as a parameter for assessing the level of polymorphism within specific populations. Pi reveals the extent of variation in nucleic acid sequences among different species. Regions with higher variability can provide potential molecular markers for population genetics (Raman et al., 2022). To identify mutation hotspots and genes in the chloroplast genome of the genus *Solanum*, we employed CLC Genomics Workbench v.20.0.3 for whole chloroplast genome alignment. We utilized DNAsp v.6.12.03 software to compute Pi. After importing the data, we adjusted the format to Haploid and Chloroplast, with a window length set to 400 and a step size of 200, in order to construct a line graph of polymorphic sites.

#### 2.8.4 Selection pressure analysis

Selection pressure refers to the external forces acting on a species during the process of biological evolution, compelling the species to adapt to its natural environment. In genetics, the ratio ω = Ka/Ks, or dN/dS, represents the relationship between non-synonymous mutations (Ka) and synonymous mutations (Ks). Synonymous mutations are not subject to natural selection, while non-synonymous mutations are influenced by it. It is generally accepted that when ω > 1, this indicates a positive selection effect, suggesting that certain advantageous mutations are actively selected. Conversely, ω = 1 denotes neutrality, which is characteristic of neutral evolution. If 0 < ω < 1, it implies the presence of purifying selection, with smaller ω values signifying stronger negative selection pressures, leading to more conserved amino acid sequences (Sheikh-Assadi et al., 2022). We utilized CLC Genomics Workbench v.20.0.3 to extract the CDS sequences of the chloroplast genome, merging and aligning the same CDS sequences from multiple samples with *S. lycopersicum* as the reference genome. Furthermore, we employed EasyCodeML v.1.31 to calculate the Ka and Ks for all species’ chloroplast protein-coding genes, and subsequently determined the ω value using the MLWL model with default parameters (Gao et al., 2019).

#### 2.8.5 Estimation of phylogeny and divergence time

CLC Genomics Workbench v.20.0.3 was utilized to compare 52 chloroplast genomes of the genus *Solanum* and to iteratively run for 1 million generations using MrBayes v3.2.7 with the Markov Chain Monte Carlo (MCMC) method, sampling every 100 generations (Ronquist et al., 2012). The initial 25% of the phylogenetic tree results were deleted, ultimately deriving a majority-rule consensus tree. Calibration points were acquired from the TimeTree website (http://www.timetree.org/), and species divergence times were obtained by the mcmctree command in PAML v.4.10.7 (Yang, 2007).

## 3 Results

### 3.1 Ploidy analysis

**Figure 1:**
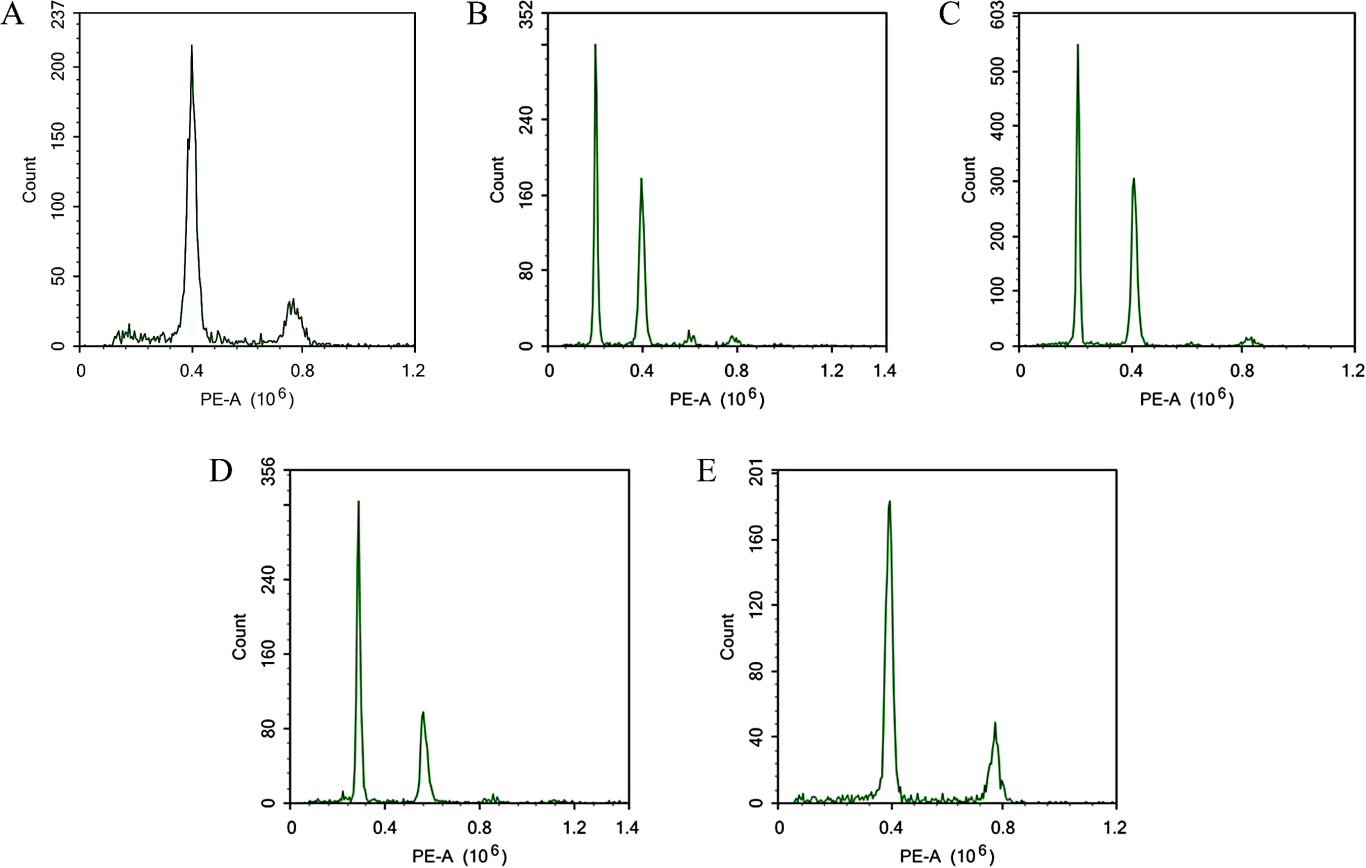
Prediction of ploidy by flow cytometry. **(A)** C88; **(B)** IVP101; **(C)** Diploid offspring; **(D)** Triploid offspring; **(E)** Tetraploid offspring.

Using flow cytometry, we analyzed the ploidy of 157 distant hybrid induction materials. The tetraploid C88 and diploid IVP101 served as controls for tetraploids and diploids, respectively. The flow cytometry results identified 43 diploids, 64 triploids, and 50 tetraploids among the 157 distant hybrid materials, representing 27.39%, 40.76%, and 31.85% of the total sample, respectively. All three purple stem materials were classified as diploids (**Figure 1**).

**Figure 2:**
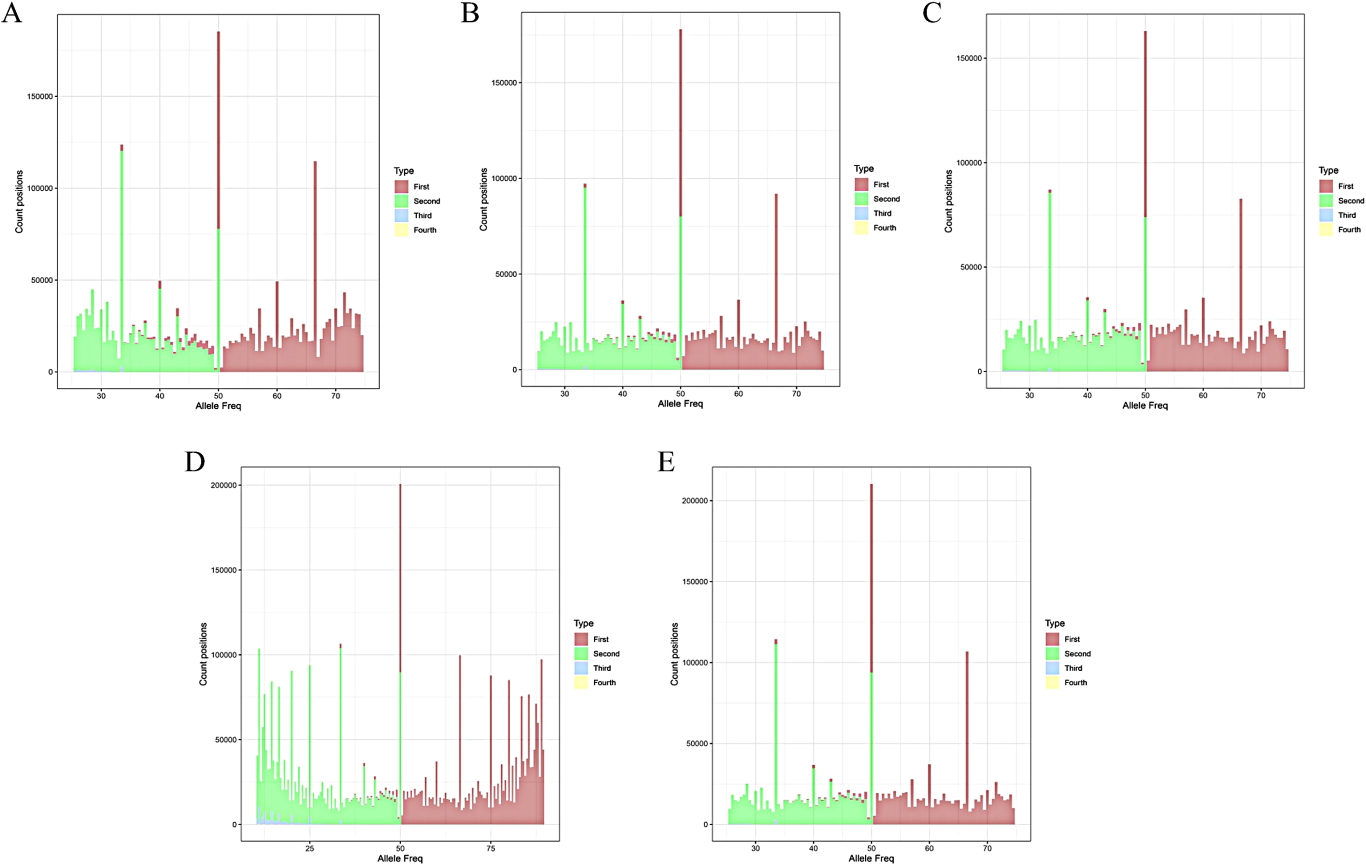
ploidy NGS histogram. **(A)** C88; **(B)** IVP101; **(C)** Diploid offspring; **(D)** Triploid offspring; **(E)** Tetraploid offspring.

To determine the ploidy of 157 offspring, we analyzed the whole-genome resequencing data using the ploidyNGS software. The findings revealed that among the 157 offsprings, 43 were diploid, 64 were triploid, and 50 were tetraploid (**Figure 2**), consistent with the results obtained from flow cytometry analysis.

### 3.2 Statistics of stem color of plants with different ploidy

The stem color of C88 is known to be green, while the stem color of inducer IVP101 is purple. Among the 157 offspring obtained from seeds without embryonic spots, 43 were diploid, 64 were triploid, and 50 were tetraploid. In the diploid population, the majority of the materials (93.02%) have green stems, but three materials have purple stems. In the triploid population, the majority of materials (85.94%) have purple stems. In the tetraploid population, 66.00% are purple and 34.00% are green (**Table 2**).

**Table 2:** Statistics of stem color in offspring materials.

Stem color	Count		
	Diploid offspring	Triploid offspring	Tetraploid offspring
Green	40 (93.02%)	9 (14.06%)	17 (34.00%)
Purple	3 (6.98%)	55 (85.94%)	33 (66.00%)
Total	43 (100%)	64 (100%)	50 (100%)

### 3.3 Identification of cytoplasmic genomic polymorphisms

**Figure 3:**
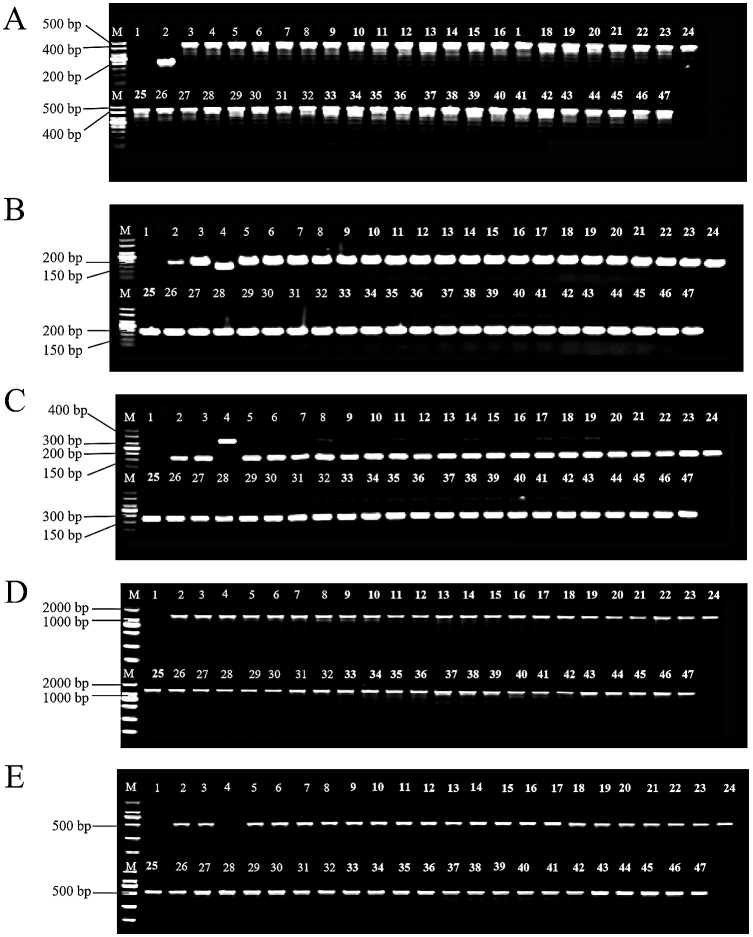
Identification of cytoplasmic genomic polymorphisms. **(A)** PCR amplification results using T-labeled primers. M: 50 bp DNA marker; 1: Blank control; 2: Atlantic; 3: C88; 4: IVP101; 5-47: Diploid offsprings; **(B)** PCR amplification results using S-labeled primers. M: 50 bp DNA marker; 1: Blank control; 2: Atlantic; 3: C88; 4: IVP101; 5-47: Diploid offsprings; **(C)** Results of *Bam*H I digestion after amplification with SAC-labeled primers. M: 50 bp DNA marker; 1: Blank control; 2: Atlantic; 3: C88; 4: IVP101; 5-47: Diploid offsprings; **(D)** Results of *Bam*H I digestion after amplification with A-labeled primers. M: 2000 bp DNA marker; 1: Blank control; 2: Atlantic; 3: C88; 4: IVP101; 5-47: Diploid offsprings; **(E)** Results of amplification with D-labeled primers. M: 2000 bp DNA marker; 1: Blank control; 2: Atlantic; 3: C88; 4: IVP101; 5-47: Diploid offsprings.

Using five sets of potato cytoplasmic marker primers, we identified the cytoplasmic types of 43 selected diploid offspring. The T marker primers amplified DNA from 46 samples. No bands appeared in the blank control, while Atlantic produced a band approximately 200 bp in size. Bands from C88 and IVP101 were around 450 bp. The 43 diploid offsprings displayed band sizes identical to the parents, all measuring 450 bp (**Figure 3A**). S primers were employed to detect polymorphisms at the chloroplast genome *rps*16/*trn*Q locus. After amplification with S primers, only IVP101 yielded a band of about 150 bp; all other materials produced bands around 200 bp (**Figure 3B**). The SAC primers were used to examine the chloroplast genome at the *1*/*11a* locus. After amplification and *Bam*H I digestion, only IVP101 produced a band of approximately 320 bp; all other materials showed bands of 175 bp (**Figure 3C**). Amplification with A primers and *Bam*H I digestion resulted in all materials yielding a band of 1700 bp (**Figure 3D**). D primers were used to assess the mitochondrial genome’s *Band1* locus, where IVP101 failed to produce any band, while Atlantic, C88, and diploid offsprings exhibited a band of roughly 500 bp (**Figure 3E**). These results indicate that the chloroplast genome of the diploid offspring at loci *trn*V-UAC/*ndh*C, *rps*16/*trn*Q, *1*/*11a*, and *10*, as well as the mitochondrial genome at the *Band1* locus, demonstrate maternal inheritance, with no observed polymorphism among the offsprings.

### 3.4 Chloroplast genome assembly and annotation

**Figure 4:**
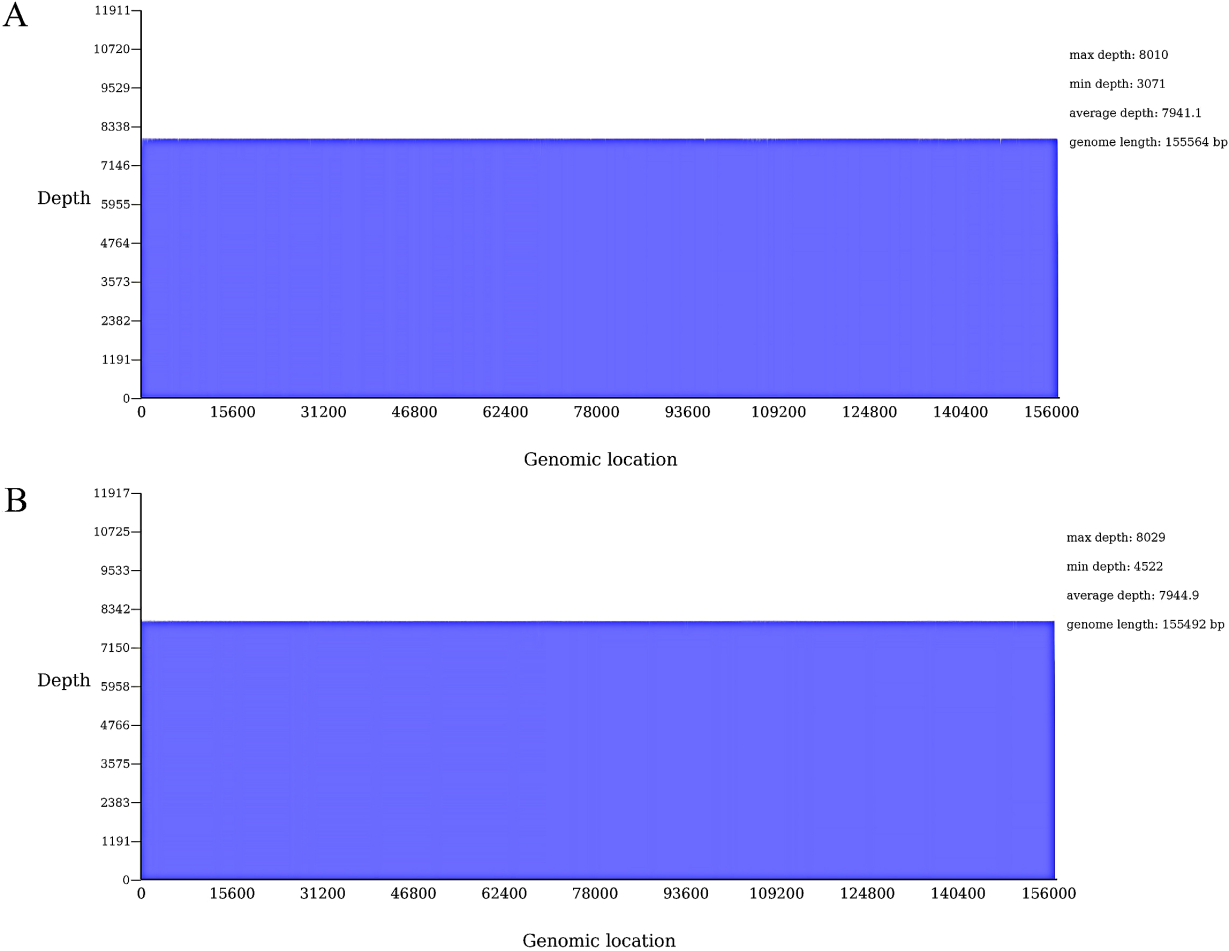
Evaluation of chloroplast genome assembly results. **(A)** C88; **(B)** IVP101.

The visualization coverage map indicates that the sequencing data uniformly covers the assembled sequence with a coverage of 100%, suggesting effective assembly (**Figure 4**).

**Figure 5:**
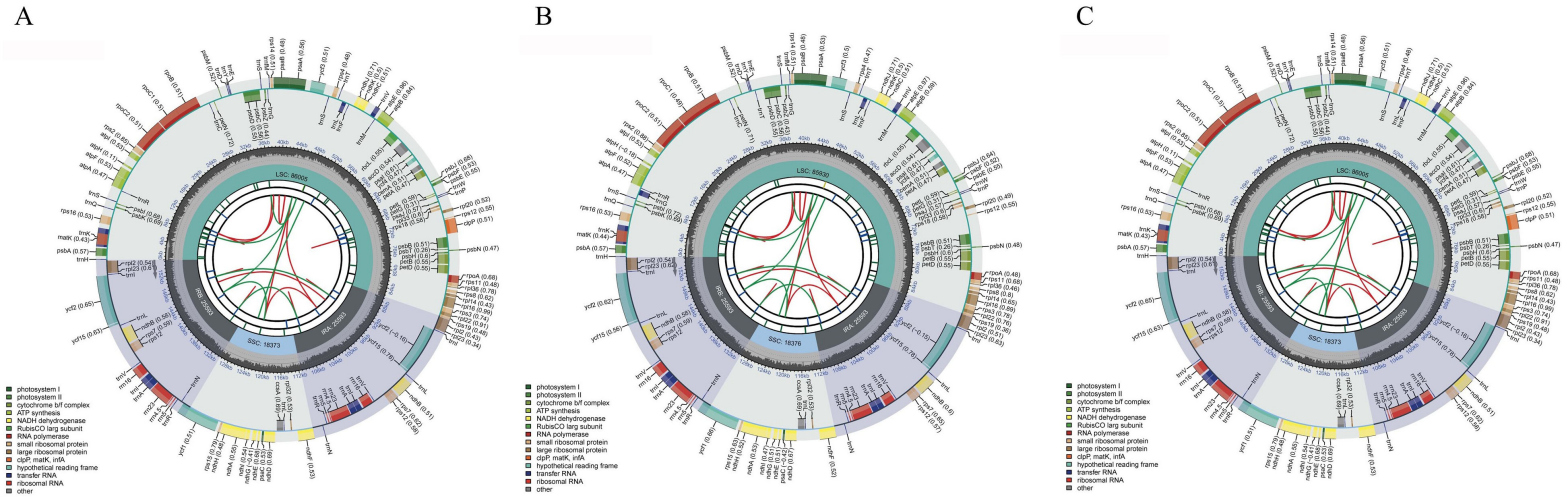
The circular maps of chloroplast genomes. **(A)** C88; **(B)** IVP101; **(C)** Diploid offspring.

Similar to the structure of chloroplast genomes in most angiosperms, the chloroplast genomes of C88, IVP101, and their diploid offspring exhibit a typical tetrad structure. This consists of a pair of single-copy regions of differing lengths and a pair of inverted repeat regions that separate these single-copy regions (**Figure 5**). The chloroplast genome lengths for C88 and IVP101 are 155,564 bp (IR: 25,593 bp; LSC: 86,005 bp; SSC: 18,373 bp) (**Figure 5A**) and 155,492 bp (IR: 25,593 bp; LSC: 85,930 bp; SSC: 18,376 bp) (**Figure 5B**), respectively. Annotation results show that the chloroplast genomes of C88 annotated 128 genes and IVP101 annotated 129 genes. There are 3 differences in the types of annotated genes. IVP101 lacks the *clp*P gene, while C88 is missing the *trn*G-UCC and *trn*T-GGU genes (**Table 3**).

**Table 3:** Gene composition of the chloroplast genome.

Gene classification	C88	IVP101	Diploid offsprings
Photosystem I	*psa*A, *psa*B, *psa*C*, psa*I, *psa*J		
Photosystem II	*psb*A, *psb*B, *psb*C, *psb*D, *psb*E, *psb*F, *psb*H, *psb*I, *psb*J, *psb*K, *psb*M, *psb*N, *psb*T, *psb*Z		
ATP synthase	*atp*A, *atp*B, *atp*E, *atp*F, *atp*H, *atp*I		
Cytochrome b/f complex	*pet*A, *pet*B, *pet*D, *pet*G, *pet*L, *pet*N		
NADH dehydrogenase	*ndh*A, *ndh*B^2^ *, ndh*C, *ndh*D*, ndh*E, *ndh*F, *ndh*G, *ndh*H, *ndh*I, *ndh*J, *ndh*K		
Rubisco	*rbc*L		
Transcription	*rpo*A, *rpo*B, *rpo*C1, *rpo*C2		
Ribosomal proteins(LSU)	*rpl*2*^2^*, *rpl*14, *rpl*16, *rpl*20, *rpl*22, *rpl*23*^2^*, *rpl*32, *rpl*33, *rpl*36		
Ribosomal proteins(SSU)	*rps*2, *rps*3, *rps*4, *rps*7^2^, *rps*8, *rps*11, *rps*12^2^, *rps*14, *rps*15, *rps*16, *rps*18, *rps*19		
Transfer RNA	*trn*A-UGC^2^, *trn*C-GCA, *trn*D-GUC, *trn*E-UUC, *trn*F-GAA, *trn*fM-CAU, *trn*G-GCC, *trn*H-GUG, *trn*I-CAU^2^, *trn*I-GAU^2^, *trn*K-UUU, *trn*L-CAA^2^, *trn*L-UAA, *trn*L-UAG, *trn*M-CAU, *trn*N-GUU^2^, *trnP-UGG*, *trn*Q-UUG, *trn*R-ACG^2^, *trn*R-UCU, *trn*S-GCU, *trn*S-GGA, *trn*S-UGA, *trn*T-UGU, *trn*V-GAC^2^, *trn*V-UAC, *trn*W-CCA, *trn*Y-GUA		
	*—*	*trn*G-UCC	*—*
	*—*	*trn*T-GGU	*—*
Ribosomal RNA	*rrn*16^2^, *rrn*23^2^, *rrn*4.5^2^, *rrn*5^2^		
Subunits of Acetyl-CoA-carboxylase	*acc*D		
Protease	*clp*P	*—*	*clp*P
Maturase K	*mat*K		
C-type cytochrome synthesis	*ccs*A		
Carbon metabolism	*cem*A		
Conserved open reading frames	*ycf*1, *ycf*2^2^, *ycf*3, *ycf*4, *ycf*15^2^		

**Figure 6:**
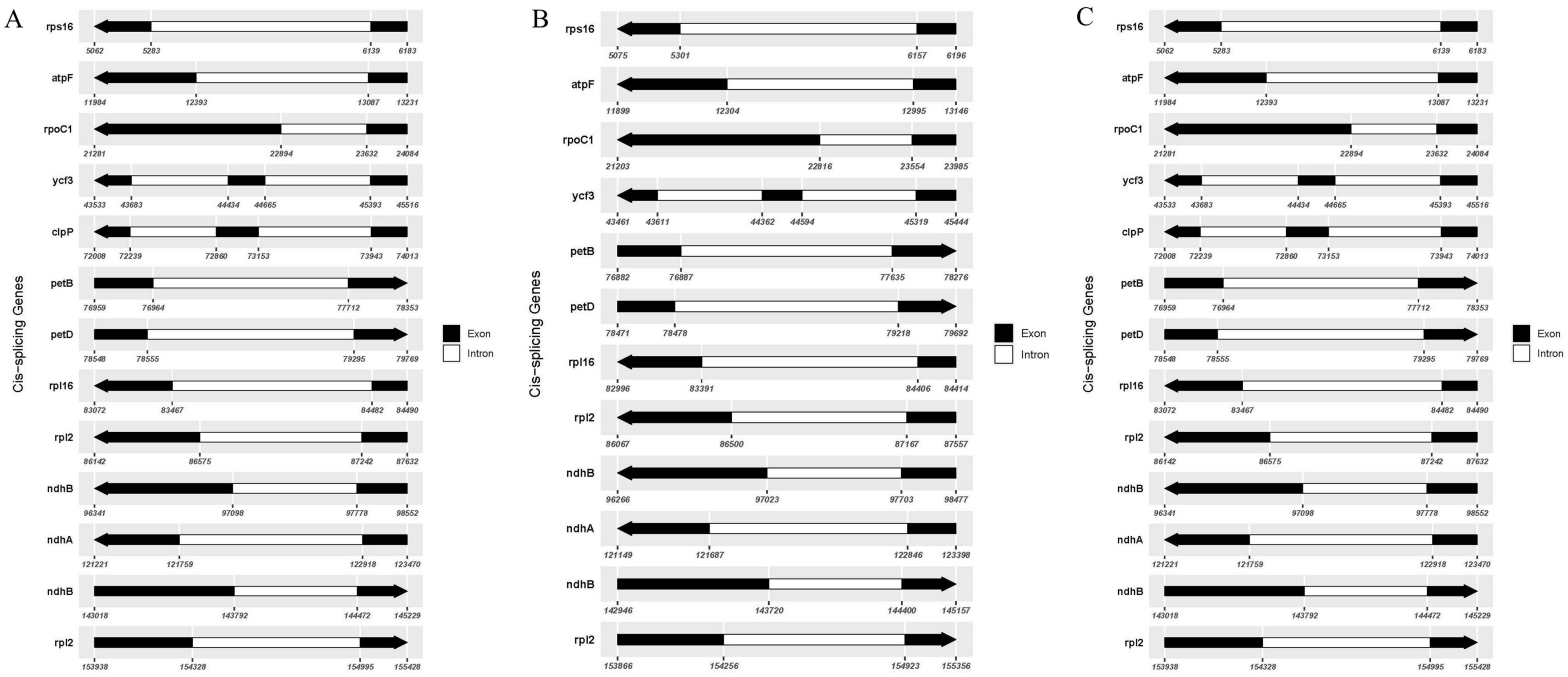
Cis-splicing genes in chloroplast genomes. **(A)** C88; **(B)** IVP101; **(C)** Diploid offspring.

**Figure 7:**
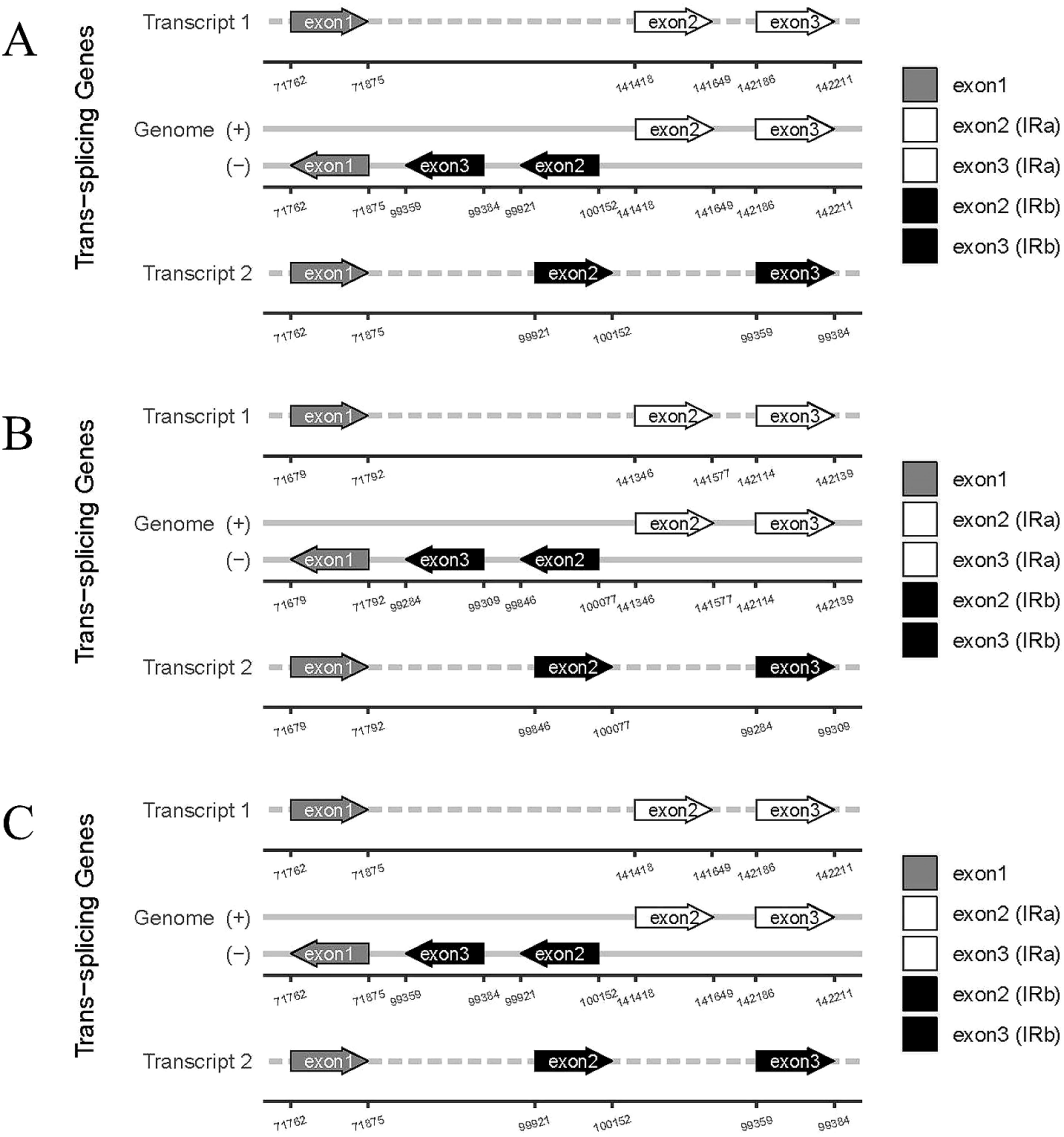
Trans-splicing genes in chloroplast genomes. **(A)** C88; **(B)** IVP101; **(C)** Diploid offspring.

The cis-splicing genes and trans-splicing genes, along with their introns and exons, are also illustrated in **Figures 6** and **7**. In the C88 chloroplast genome, 13 cis-splicing genes were identified. Among these, *rps*16, *atp*F, *rpo*C1, *pet*B, *pet*D, *rpl*16, *rpl*2 (×2), *ndh*B (×2), and *ndh*A each contained one intron, while *ycf*3 and *clp*P each shared two introns (**Figure 6A**). In contrast, The IVP101 chloroplast genome predicted 12 cis-splicing genes, containing *rps*16, *atp*F, *rpo*C1, *pet*B, *pet*D, *rpl*16, *rpl*2 (×2), *ndh*B (×2), *ndh*A includes one intron, while *ycf*3 consists of two introns (**Figure 6B**).

The *rps*12 gene represents a trans-splicing gene, features three exons. The white region denotes the second exon of IRa, the black region represents the second exon of IRb, and the gray area indicates the first exon. The 5′ end is located in the LSC region, whereas the 3′ end lies in the IR region (**Figure 7**). The chloroplast genome size, annotated gene count and types, cis-splicing genes, trans-splicing genes, and their intronic and exonic structures in 43 diploid offspring completely aligns with the C88 findings.

### 3.5 Detection of variant loci in chloroplast genomes

Using the C88 chloroplast genome as a reference, we analyzed the variation sites in the chloroplast genomes of inducer IVP101 and all diploid offsprings. The results indicated that the chloroplast genome sequence in all diploid offsprings showing no detectable variation sites. In contrast, the chloroplast genome sequence of inducer IVP101 revealed 26 insertion sites, 21 deletion sites, and 170 single nucleotide mutation sites (**Table 4**; **Supplementary Tables 2, 3**).

**Table 4:** Chloroplast genome variation sites.

Types of mutation loci		IVP101	Diploid offsprings
InDels	Insertion	26	0
	Deletion	21	0
SNPs	Conversion	75	0
	Transposition	95	0

### 3.6 Analysis of nuclear genome introgression

To further understand if introgression occurred crossing nuclear and chloroplast in purple stem diploid offsprings, we utilize Dsuite to estimate the theoretical introgression value of the inducer IVP101 (H3) nuclear genome. The D statistic ranges from [-1, 1]. D statistic of 0 indicates no gene flow occurs; if gene exchange occurs between H2 and H3, the D statistic exceeds 0; if gene exchange occurs between H1 and H3, the D statistic drops below 0 (Malinsky et al., 2021). The estimation results indicate that ABBA is 6,100.0, BABA is 5,810.5, and the D statistic is 0.0243063. This finding suggests gene exchange occurred between the purple-stem diploid offsprings and the inducer IVP101 (**Table 5**).

**Table 5:** Estimation of theoretical introgression value between groups.

H1	H2	H3	Dstatistic	BBAA	ABBA	BABA
Green-stem diploid offsprings	Purple-stem diploid offsprings	IVP101	0.0243063	10,754.2	6,100.0	5,810.5

**Figure 8:**
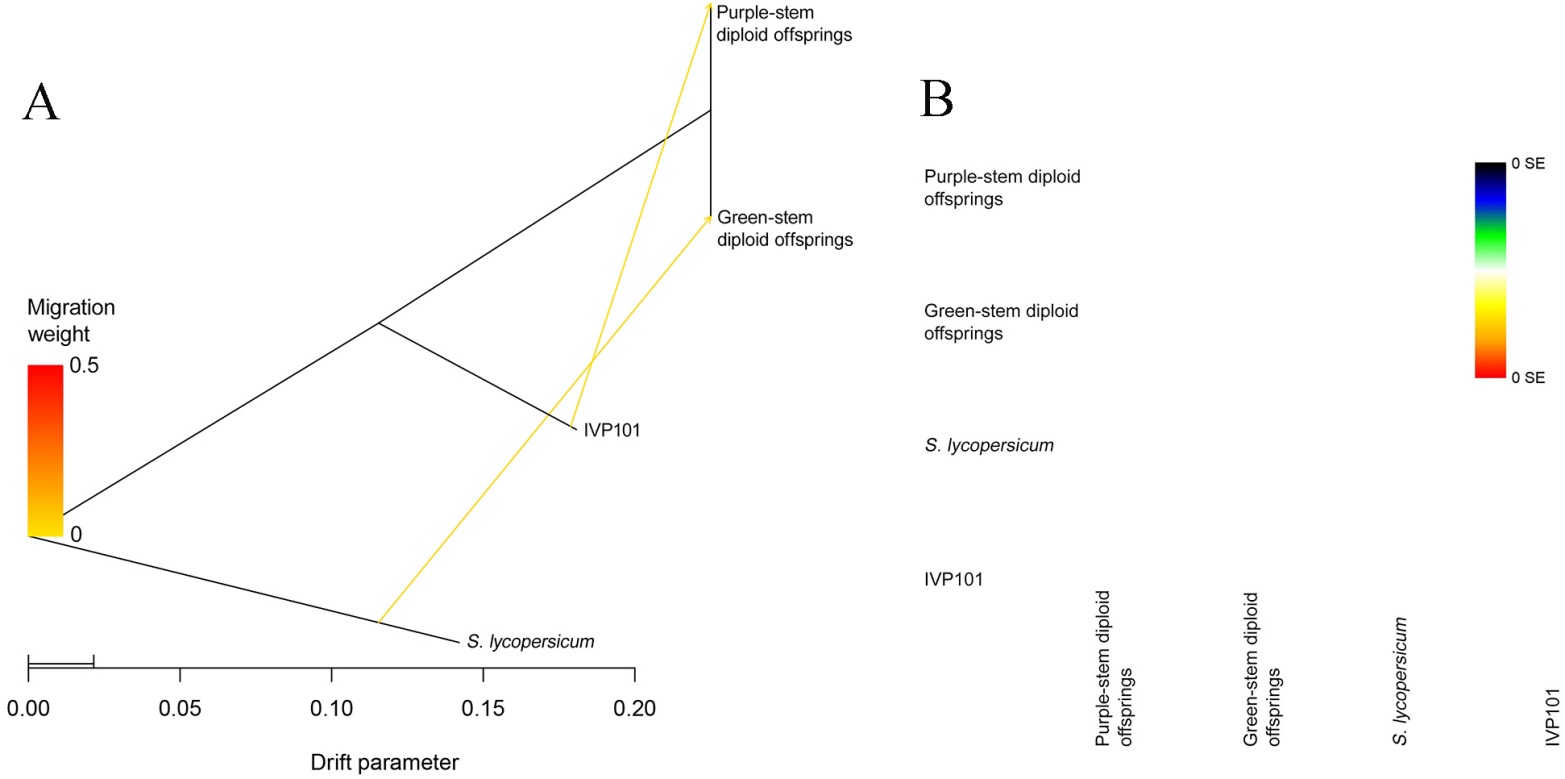
Inference of gene introgression direction. **(A)** Treemix maximum likelihood tree. The scale bar below shows the 10 times average standard deviation of the elements in the sample covariance matrix; **(B)** Residual fit heat map. On the right is the color scale. The residuals above white (0 points) indicate that the relationship between the corresponding populations is closer than that on the maximum likelihood tree, suggesting that there are gene introgression events between these populations.

The TreeMix analysis also indicates that the tree includes two arrows. One arrow points from *S. lycopersicum* to the green-stem diploid offsprings, while the second arrow directs from the inducer IVP101 to the purple-stem diploid offsprings (**Figure 8A**). In the accompanying heatmap scale, 0 SE represents the topmost color, suggesting that the model fits well with the covariance among actual populations (**Figure 8B**). This finding implies that the nuclear genomic introgression direction proceeds from the inducer IVP101 to the purple-stem diploid offsprings.

### 3.7 Comparative analysis of the chloroplast genomes of the genus *Solanum*

#### 3.7.1 Comparative analysis of the chloroplast genome characteristics

The chloroplast genomes of 14 *Solanum* species exhibit a typical tetrad structure. The total size of the chloroplast genomes ranges from 154,289 bp to 155,614 bp, with an average size at 155,369 bp. The LSC region spans from 84,748 bp to 86,029 bp, averaging at 85,797 bp. The SSC region varies between 18,347 bp and 18,420 bp, with an average at 18,372 bp. The IR regions measured between 25,563 bp and 25,628 bp, averaging 25,600 bp. The GC content of the chloroplast genomes remains consistent at 37.90%, while the number of annotated genes ranges from 128 to 141 (**Table 6**).

**Table 6:** Basic characteristics of chloroplast genome in *Solanum*.

Species	Total size/bp	LSC /bp	IR /bp	SSC /bp	Total genes	Protein coding genes	tRNA genes	rRNA genes	Overall GC content/%
*S. lycopersicum*	155,461	85,882	25,608	18,363	133	87	37	8	37.9
*S. melongena*	154,289	84,748	25,563	18,420	133	85	39	8	37.9
*S. tuberosum*	155,296	85,737	25,593	18,373	141	84	45	8	37.9
Desiree									
*S. verrucosum*	155,479	85,946	25,593	18,347	130	85	37	8	37.9
*S. pimpinellifolium*	155,442	85,856	25,612	18,362	128	83	37	8	37.9
*S. pennellii*	155,254	85,680	25,613	18,348	134	87	37	8	37.9
*S. americanum*	155,266	85,658	25,615	18,378	135	83	37	8	37.9
*S. commersonii*	155,525	85,973	25,593	18,366	133	86	39	8	37.9
*S. habrochaites*	155,465	85,877	25,612	18,364	128	83	37	8	37.9
*S. pinnatisectum*	155,614	86,029	25,595	18,395	130	85	37	8	37.9
*S. stenotomum*	155,492	85,930	25,593	18,376	130	85	37	8	37.9
*S. chilense*	155,528	85,907	25,628	18,365	128	83	37	8	37.9
*S. phureja* IVP101	155,492	86,005	25,593	18,373	130	85	37	8	37.9
*S. tuberosum* C88	155,564	85,930	25,593	18,376	130	86	36	8	37.9

#### 3.7.2 Characteristics of the SC/IR boundary

**Figure 9:**
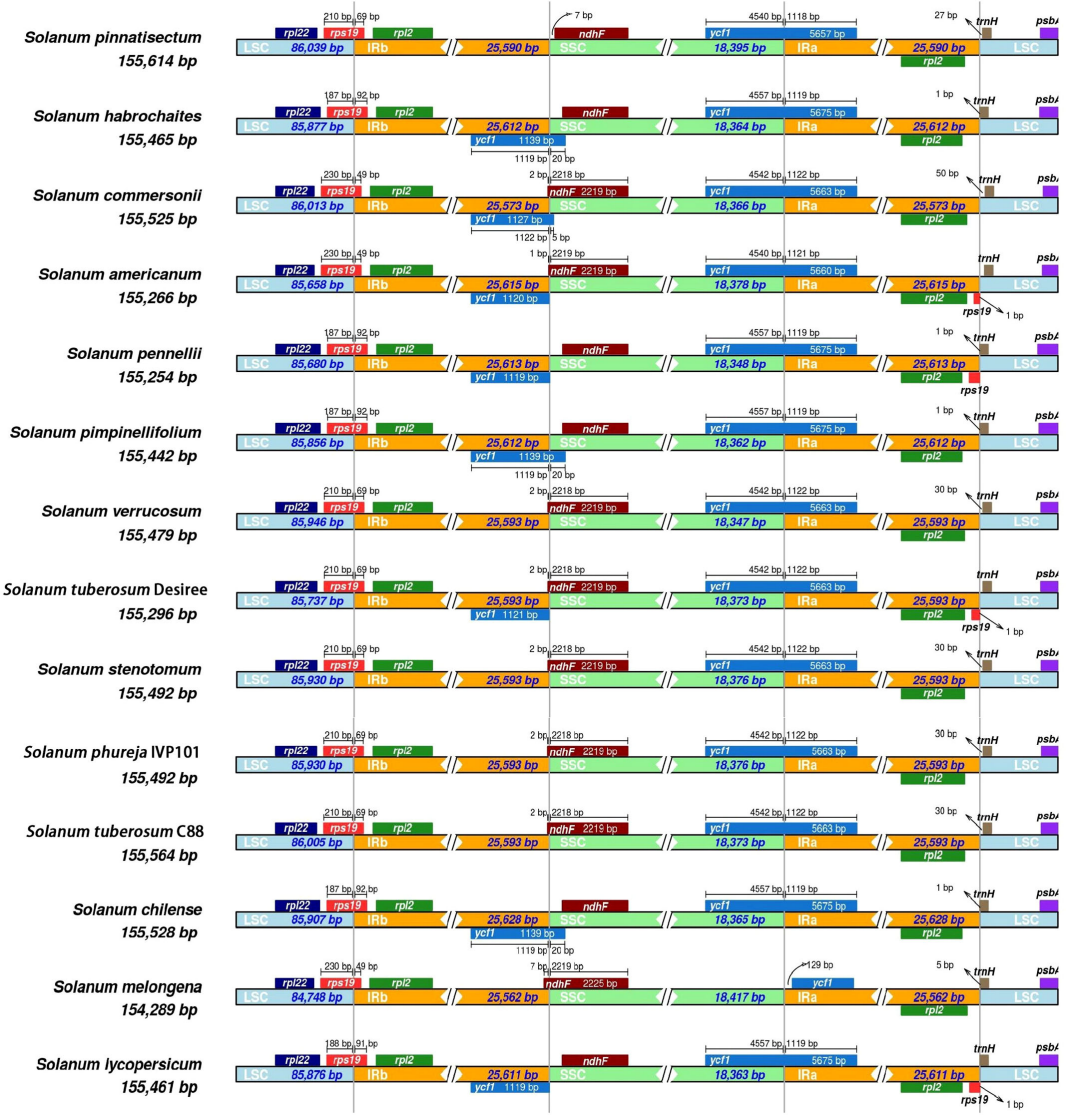
SC/IR boundary of *Solanum* chloroplast genome.

By comparing the SC/IR boundary regions of the chloroplast genomes in *Solanum* species, we observe that while the length of the IR regions remains relatively consistent across 14 species, notable variations exist in their SC/IR boundaries (**Figure 9**). The *rps*19 gene, measuring 279 bp, spans the LSC/IRb regions of all 14 *Solanum* chloroplast genomes. However, the length of *rps*19 exhibits dynamic changes in the LSC and IRb regions: for six species, *rps*19 is 210 bp in the LSC and 69 bp in the IRb; for four species, it is 187 bp in the LSC and 92 bp in the IRb; for three species, it is 230 bp in the LSC and 49 bp in the IRb; finally, *S. lycopersicum* displays an *rps*19 length of 188 bp in the LSC and 91 bp in the IRb. Except for *S. melongena*, *ycf*1 entirely resides within the IRa region, positioned 129 bp from the boundary. The *ycf*1 gene crosses the SSC/IRa boundary in the remaining 13 *Solanum* species’ chloroplast genomes, overlapping at the IRa boundary. The overlapping segments range in length from 1,118 bp to 1,122 bp. These incomplete overlapping fragments lead to the formation of *ycf*1 pseudogenes at the IRb/SSC boundaries in eight species. The *ndh*F gene lies at the IRb/SSC boundary and spans this boundary in all eight species. The lengths in the IRb and SSC regions measure 1-7 bp and 2,218-2,219 bp, respectively. Notably, in *S. commersonii*, *S. americanum*, and *S. tuberosum* Desiree, the *ndh*F gene overlaps with the *ycf*1 pseudogene. In the other six species, *ndh*F does not cross the IRb/SSC boundary but is fully contained within the SSC region; for instance, the *ndh*F gene in *S. pinnatisectum* is situated 7 bp from the boundary. In 14 species of the genus *Solanum*, the *trn*H gene was detected at the IRa/LSC boundary, completely located within the LSC region, at a distance of 0-50 bp from the boundary. In *S. americanum*, *S. pennellii*, *S. tuberosum* Desiree, and *S. lycopersicum*, the *rps*19 gene was also found at the IRa/LSC boundary. However, due to incomplete copies, a pseudogene of *rps*19 formed in the IRa region.

#### 3.7.3 Analysis of mutation hotspots and genetic selection pressure

**Figure 10:**
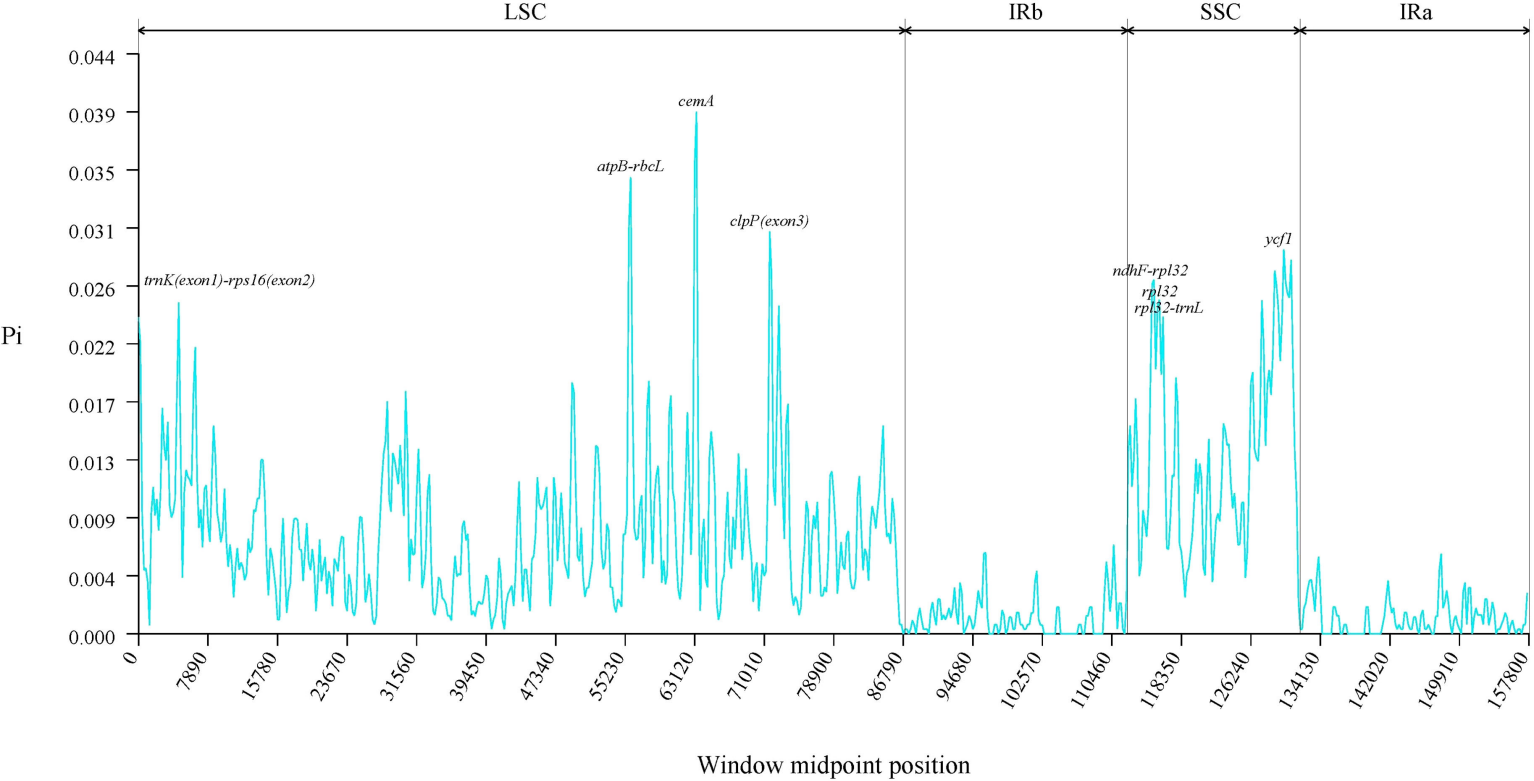
The nucleotide diversity of chloroplast genomes within the genus *Solanum*.

To assess the sequence variation among the chloroplast genomes of 14 *Solanum* species, we calculated their Pi values. Results indicated a range from 0 to 0.03937, with the highest Pi value of 0.03937 located at the *cem*A gene in the LSC region (**Supplementary Table 4**). The analysis identified three hotspots where 0.030 ≤ Pi < 0.040, specifically in the *atp*B-*rbc*L intergenic region, *clp*P (exon 3), and the *cem*A gene region. Additionally, five hotspots showed 0.025 ≤ Pi < 0.030, located in the *ndh*F-*rpl*32, *rpl*32-*trn*L, *trn*K (exon 1)-*rps*16 (exon 2) intergenic regions, along with the *ycf*1 and *rpl*32 gene regions. Both the LSC and SSC regions emerged as zones of high variability. These highly variable areas may harbor information on rapidly evolving loci and could serve as potential molecular markers (**Figure 10**).

**Figure 11:**
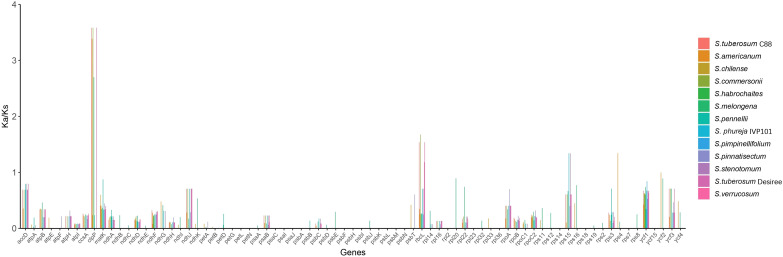
Selection pressure on chloroplast genomes of 14 *Solanum* species.

Analysis of gene selection pressures reveals that within the chloroplast genomes of 14 *Solanum* species, the genes *clp*P, *rbc*L, *rps*15, and *rps*4 exhibit signs of positive selection, while 52 genes are subject to purifying selection (**Figure 11**).

#### 3.7.4 Phylogenetic analysis and divergence time estimation

**Figure 12:**
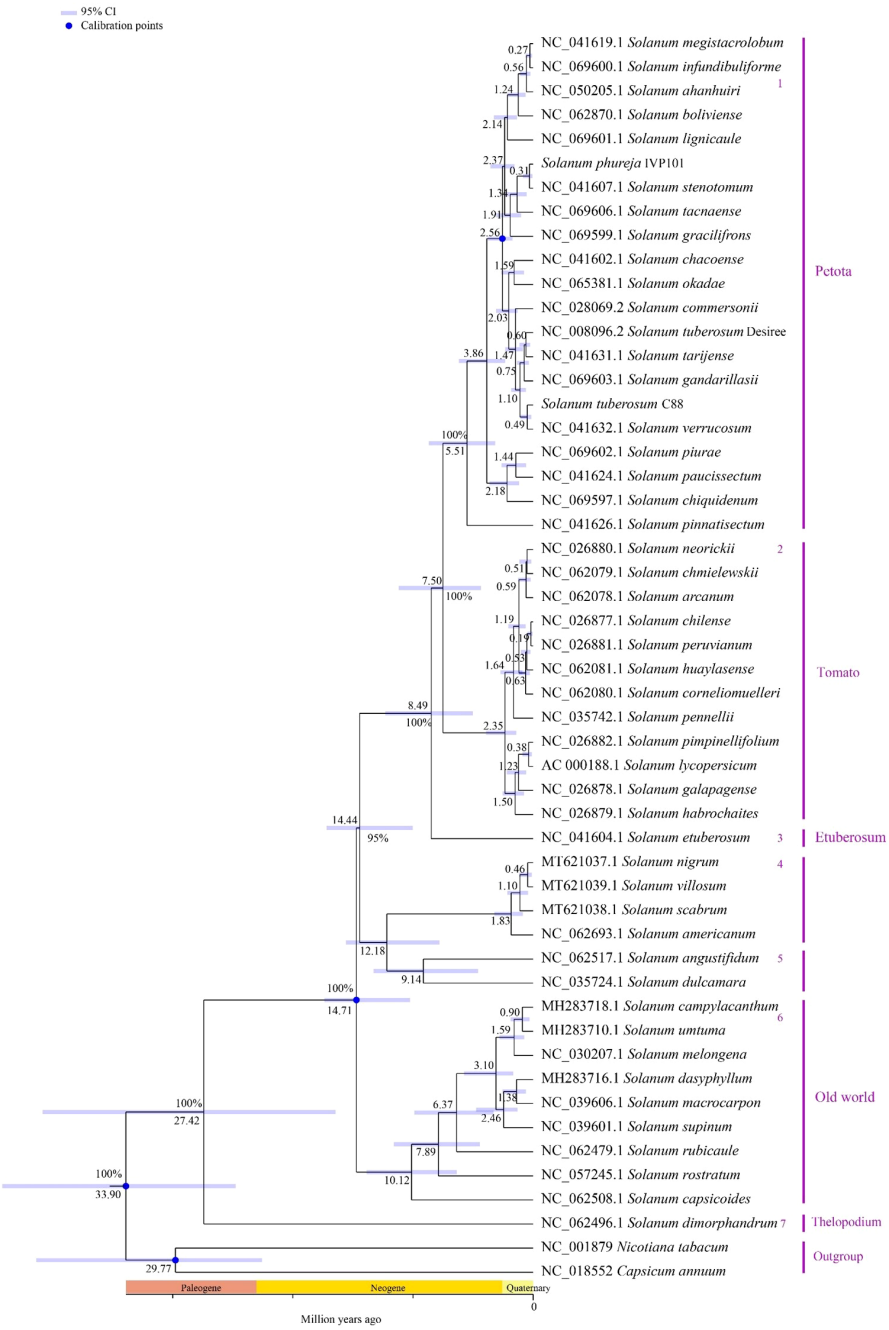
Phylogenetic relationships and divergence time estimation among 52 *Solanum* species. The numbers at nodes represent the median divergence times, with blue lines indicating the confidence intervals (95%) of estimated divergence times for each node; blue circles denote fossil-calibrated positions; the numbers 1-7 on the right side indicate branch numbers, and the text indicates individual species names and the names of the branches to which they belong.

Using the chloroplast genome data of the *Solanum* genus published in the NCBI database, and employing *Capsicum annuum* and *Nicotiana tabacum* as outgroups, we constructed a phylogenetic tree for the *Solanum* genus using Bayesian methods. The results indicate that among the 52 species of *Solanum*, *S. dimorphandrum* forms a monophyletic group with 100% support, diverging approximately 27.42 million years ago. The remaining 51 *Solanum* species diverged about 14.71 million years ago into three primary branches. The first branch comprises Petota, Tomato, and Etuberosum, with Etuberosum being a non-tuber-bearing entity, diverging around 8.49 million years ago, while Petota and Tomato diverged later. The second branch includes *S. nigrum*, *S. villosum*, *S. scabrum*, *S. americanum*, *S. angustifidum*, and *S. dulcamara*. The third branch mainly consists of species from the Old World lineage (**Figure 12**).

## 4 Discussion

Potatoes serve as a crucial agricultural crop. Their cytoplasmic types significantly impact the enhancement of resistance, adaptability, and other essential traits. The cytoplasmic types of potatoes are determined by both cpDNA and mtDNA. This study employed five pairs of potato cytoplasmic marker primers, comprising four pairs of cpDNA markers and one pair of mtDNA markers, to assess the cytoplasmic polymorphisms in 43 diploid materials generated from the distant hybridization of C88 and IVP101. The results obtained from the five primer pairs aligned with expectations, confirming that all diploid offspring exhibited cytoplasmic types consistent with the C88 parent. The assembly results of the chloroplast genome indicated that all diploid offspring exhibited an identical chloroplast genome sequence to the C88 maternal line. This finding suggests that the diploid offsprings display an absolute maternal inheritance pattern for chloroplast genomes. Li et al. investigated the chloroplast genetic patterns of four *Solanum* hybrids: *S. melongena* × *S. aethiopicum*, *S. melongena* × *S. torvum*, *S. aethiopicum* × *S. melongena*, and *S. aethiopicum* × *S. aethiopicum* (Li et al., 2021); They found that the chloroplast genome of the hybrids *S. melongena* × *S. aethiopicum* and *S. aethiopicum* × *S. aethiopicum* was identical to their maternal species. In contrast, the LSC region of the chloroplast genome in the hybrid *S. melongena* × *S. torvum* was 2 bp longer than that of its maternal line. Furthermore, compared to the maternal species, the LSC, IR, and SSC regions of the hybrid *S. aethiopicum* × *S. melongena* were shortened by 4 bp, 1 bp, and 2 bp, respectively (Li et al., 2021). The chloroplast genome of the *Helianthus* genus also exhibits typical maternal inheritance (Wills et al., 2005). However, in 323 distantly hybridized offspring of *Helianthus verticillatus*, 1.86% of the progeny displayed paternal lineage-derived chloroplast genomes (Ellis et al., 2008). Similar instances have also been observed in the genus *Daucus* (Boblenz et al., 1990) and Medicago (Masoud et al., 1990). Some researches suggest that even in plants where chloroplast inheritance primarily follows a maternal pattern, hybrid offspring may exhibit low frequencies of paternal inheritance (not exceeding 5%) when the number of descendants is sufficiently large (Chung et al., 2023). Possible mechanisms contributing to this phenomenon include: (1) individual chloroplasts may be allocated to reproductive cells during the first division of pollen, which then merge with the sperm to form the zygote; (2) nutritive cells from the paternal parent may also enter the female gamete, leading to the presence of chloroplast genomic material from the paternal lineage in the offspring; (3) both situations may occur simultaneously (Chung et al., 2023).

The inducer IVP101 used in this study is a homozygous individual with dominant purple embryo spot trait. In the offspring materials obtained from seeds without embryonic spots, the numbers of diploid, triploid, and tetraploid were 43, 64, and 50, respectively, with a diploid induction rate of 27.39%. The induction rate of diploids is influenced by the maternal genotype (Liu and Douches, 1993). The majority of diploid materials (93.02%) have green stems, which may have developed from 2n gametes of C88. However, some diploid materials (6.98%) have purple stems, indicating the possible involvement of the inducer (IVP101) genome in the genome of these materials. The results of nuclear genome introgression analysis further confirmed this hypothesis, that is, the IVP101 nuclear genome infiltrated into the nuclear genome of the diploid offsprings resulting in purple stem. Previous studies have suggested that diploidy is caused by 2× sperm to fertilize the central cell without fertilizing the egg, and then the egg is parthenogenetically developed, or the sperm fertilizes the egg, but the sperm genome is completely eliminated after successful fertilization (Amundson et al., 2020). In these two mechanisms, the inducer does not contribute any of its own genetic material. However, some studies have reported specific DNA markers from inducers in diploid offspring. For example, Amundson et al. found genetic materials from inducers in 8 dihaploid nuclear genomes induced by IVP101, IVP35, and PL-4 (Amundson et al., 2021). Among these, 6 had complete chromosomes, while 2 had large fragments of chromosomes. In addition, they also detected the infiltration of smaller DNA fragments from inducers. Among the 95 dihaploids produced by the hybridization of potato tetraploid variety Superior and IVP101, vt_sup_h27, vt_supp_h38 and vt_sup_h57 have extensive purple pigmentation in leaves and stems, and more than 85% of the IVP101-specific alleles in these three progenies are heterozygous (Pham et al., 2019). The possible mechanism of producing diploid offspring carrying inducer-specific genetic markers is the incomplete elimination of the inducer ‘s genome after fertilization (Zhang et al., 2008; Li et al., 2017). If all the genetic materials of the inducer are not eliminated occasionally, it is expected that the complete chromosome, chromosome fragment or DNA fragment of the inducer will be integrated into the maternal chromosome (introgression) and inherited to the offsprings. Therefore, in this study, the offspring that did not express purple spot markers, flow cytometry and ploidyNGS analysis showed diploids may be true dihaploids (free of pollinator genome) produced by parthenogenesis of C88, or diploids produced by complete elimination of the inducer genome in the zygote (without pollinator genome), which can be useful for further breeding research.

This study presents the first complete assembly of the chloroplast genomes of C88 and IVP101, followed by a comparative analysis with the chloroplast genomes of twelve other *Solanum* species. The chloroplast genome of IVP101 lacks the *clp*P gene, which is also absent in the reference genome *S. phureja* voucher PI 195191 (NC_041625.1). The *clp*P gene plays a crucial role in degrading abnormal or damaged proteins within the chloroplast. Its absence may lead to the accumulation of malfunctioning proteins, subsequently impacting the functionality and homeostasis of the chloroplast (Majeran et al., 2019). In plants with abnormal *clp*P gene function, the leaf surface becomes rough due to clumping, and the expansion of lateral leaves is irregularly prevented, resulting in asymmetric elongated leaf shapes (Shikanai et al., 2001). The ultrastructural analysis of tobacco chloroplast development shows that *clp*P disruption can also lead to swelling of meristematic plastid vesicles and inhibit chloroplast development in the dark (Shikanai et al., 2001). The C88 chloroplast genome lacks the *trn*G-UCC and *trn*T-GGU genes. These genes code for glycine and threonine tRNA, respectively. Although the *trn*G-UCC and *trn*T-GGU genes themselves do not directly control the phenotype of plants, as key tRNA genes, they have indirect effects on plant growth and phenotype development (Lorenzana and Rico, 2024). Glycine is involved in many metabolic pathways, including photosynthetic phosphorylation and chlorophyll synthesis. If the function of the *trn*G-UCC gene is affected, it will affect the color, morphology, and overall health of plants, while plants with abnormal function of the *trn*T-GGU gene will exhibit adverse phenotypes such as slow growth and yellowing of leaves (Abdullah et al., 2021). In addition, we did not find any leaf deformities or yellowing in C88 and IVP101 plants as mentioned above, this may be due to two reasons: firstly, the transfer of chloroplast genes to the nucleus or mitogenome during nuclear-cytoplasmic interaction to exert their functions in some potato varieties; secondly may be the incomplete CDS of *clp*P on genome, which were fragmented or contained introns, and not annotated by CPG packages.

Eight chloroplast genomes of the *Solanum* genus exhibited an incomplete copy of the *ycf*1 pseudogene at the IRb/SSC boundary. Additionally, *S. americanum*, *S. pennellii*, *S. tuberosum* Desiree, and *S. lycopersicum* formed the *rps*19 pseudogene at the IRa/LSC boundary, thereby enhancing the genetic diversity of the chloroplast genomes within the *Solanum* genus. Pseudogenization is common in chloroplast genomes, often caused by mutations such as base substitutions, insertions, or deletions that result in the appearance of premature stop codons in coding regions. In the subfamily Commelinoideae, pseudogenization had been observed in *acc*D, *rpo*A, and *ycf*15, caused by base insertions or deletions, while pseudogenization of *ndh*B in *Pollia japonica* Thunb. and *Rhopalephora scaberrima* (Blume) Faden was due to point mutations (Jung et al., 2021). Another case of pseudogenization occurs when coding regions are truncated, usually at the boundary regions. For example, *ycf*1 in *Aconitum carmichaelii* (Debeaux) and *Aconitum coreanum* (Lévl.) Rapaics was located at the boundary between IRA and SSC, leading to its pseudogenization (Park et al., 2017). The length of the IR regions among the 14 *Solanum* species ranged from 25,563 bp to 25,628 bp, with an average of 25,600 bp, indicating no significant difference, suggesting that the IR regions of *Solanum* species had not undergone significant expansion or contraction. Many studies had demonstrated that gene loss or pseudogenization in the chloroplast genome was often associated with the transfer of functional genes to the nuclear genome, resulting in their substitution by nuclear-encoded proteins (Wicke et al., 2011; Nevill et al., 2019). For example, *inf*A had undergone multiple independent transfers to the nucleus (Millen et al., 2001), and in *Hypericum ascyron*, *inf*A, *rps*7, *rps*16, *rpl*23, and *rpl*32 genes had all been transferred to the nuclear genome (Claude et al., 2022).

To date, over 20 regions within the chloroplast genomes of plants have been proposed as loci for phylogenetics, species delimitation, and barcoding, including *mat*K, *rbc*L, *trn*H-*psb*A, and *ycf*1 (Shaw et al., 2014). Through sliding window analysis, we identified eight divergence hotspots located in the single-copy regions of the *Solanum* chloroplast genome (*atp*B-*rbc*L, *ndh*F-*rpl*32, *rpl*32-*trn*L, *trn*K-*rps*16 intergenic regions, and within the *clp*P, *cem*A, *ycf*1, and *rpl*32 gene regions), which may serve as potential DNA barcodes for *Solanum* species. Selection pressure analysis indicates that the *clp*P, *rbc*L, *rps*15, and *rps*4 genes have undergone positive selection, while most genes are under purifying selection. This finding was consistent with previous research indicating that most chloroplast genes in angiosperm species were under purifying selection, reflecting a highly conservative evolutionary history, because purifying selection helped prevent mutations and preserves the conservative functions of genes (Huang et al., 2021; Yang et al., 2020; Wu et al., 2020). The current phylogenetic relationships among the *Solanum* genus has developed into three main clades: (1) Thelopodium clade, which includes *Thelopodium crispum*, *T. pygmaeum*, and *T. schottii*; (2) Clade I consists of approximately 350 species, primarily herbaceous and non-spiny plants, including Tomato, Petota, and Basarthrum; (3) Clade II encompasses about 900 species, mainly spiny and shrubby plants, with the latter two clades further divided into 10 major clades and 43 minor clades (Stern et al., 2011; Tepe et al., 2016). Earlier studies suggested a closer evolutionary relationship between Etuberosum and Petota than with Tomato, but the results of this study indicated that Etuberosum diverged approximately 8.49 million years ago, possibly as the sister group to the common ancestor of Tomato and Petota. Tang et al.’s study indicated that Etuberosum was the sister group to the common ancestor of Tomato and Petota, diverging approximately 8.30 million years ago (Tang et al., 2022). The phylogenetic tree constructed in this study suggested that *S. dimorphandrum* was the sister group to other *Solanum* species and should be classified within the Thelopodium clade. Further investigation revealed that *S. dimorphandrum* was a species newly sampled by Gagnon et al (Gagnon et al., 2022). They also constructed a new Sanger supermatrix containing 60% of *Solanum* species, and the results of this phylogenetic study also placed *S. dimorphandrum* within the Thelopodium clade (Gagnon et al., 2022).

## Data Availability

The data provided in this study are deposited in the NCBI GenBank database (accessed on 11 January 2024). The chloroplast sequences of IVP101 and C88 had been uploaded to GenBank and the accession numbers are PP680311 and PP680310. The original contributions presented in the study are included in the article/**Supplementary Material**. Further inquiries can be directed to the corresponding authors.
