# Supplementary figures and images for: Genetic characteristics of the diploid offsprings in potato Cooperation 88 induced by diploid donor IVP101
Source: Front Plant Sci. 2024 Nov 8;15:1486549. doi: 10.3389/fpls.2024.1486549 (PMC11582670; doi:10.3389/fpls.2024.1486549)

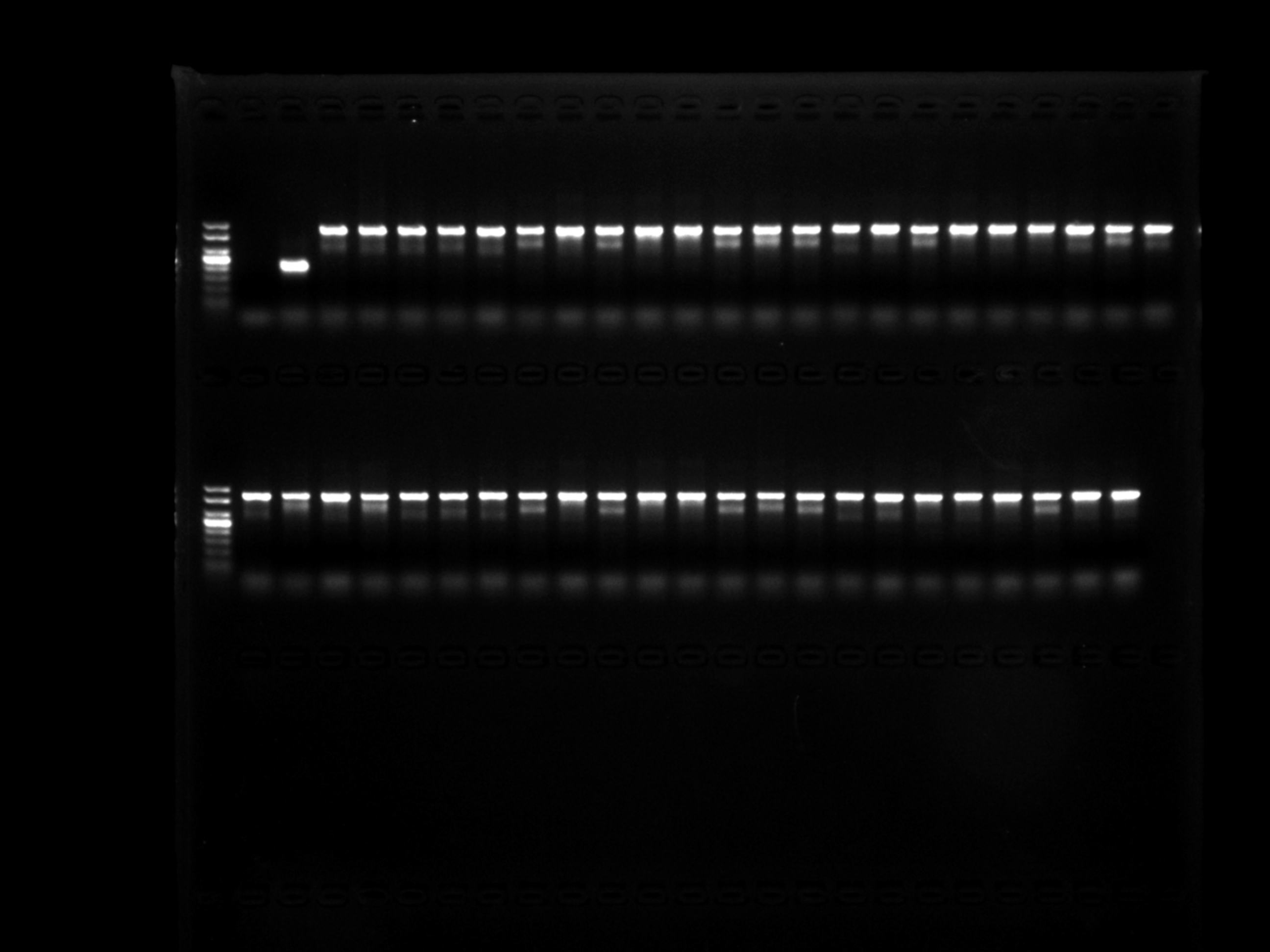

Supplement: Supplementary file 1 [file DataSheet1.zip › Original gels/Figure 3A.jpg]

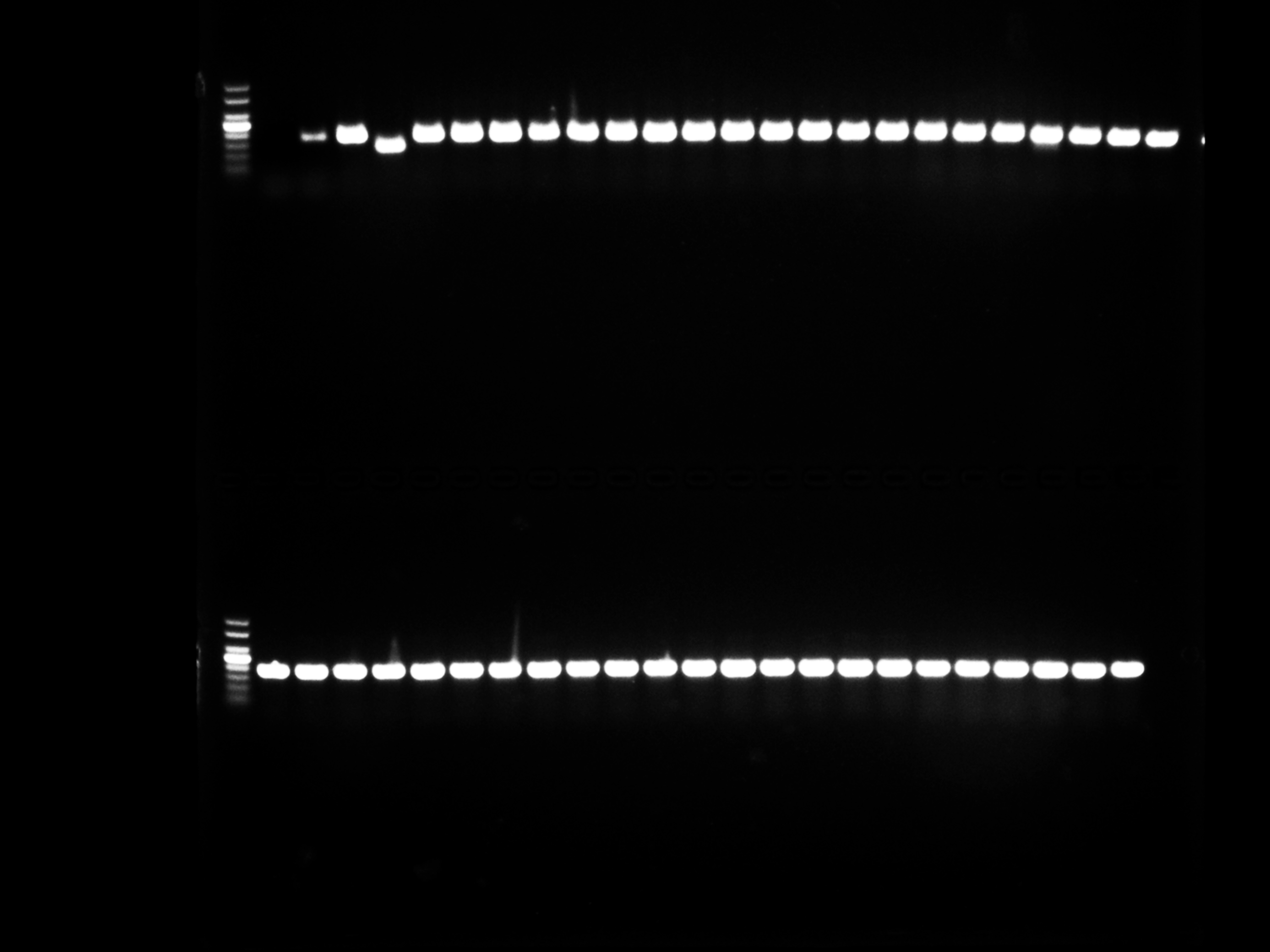

Supplement: Supplementary file 1 [file DataSheet1.zip › Original gels/Figure 3B.tif]

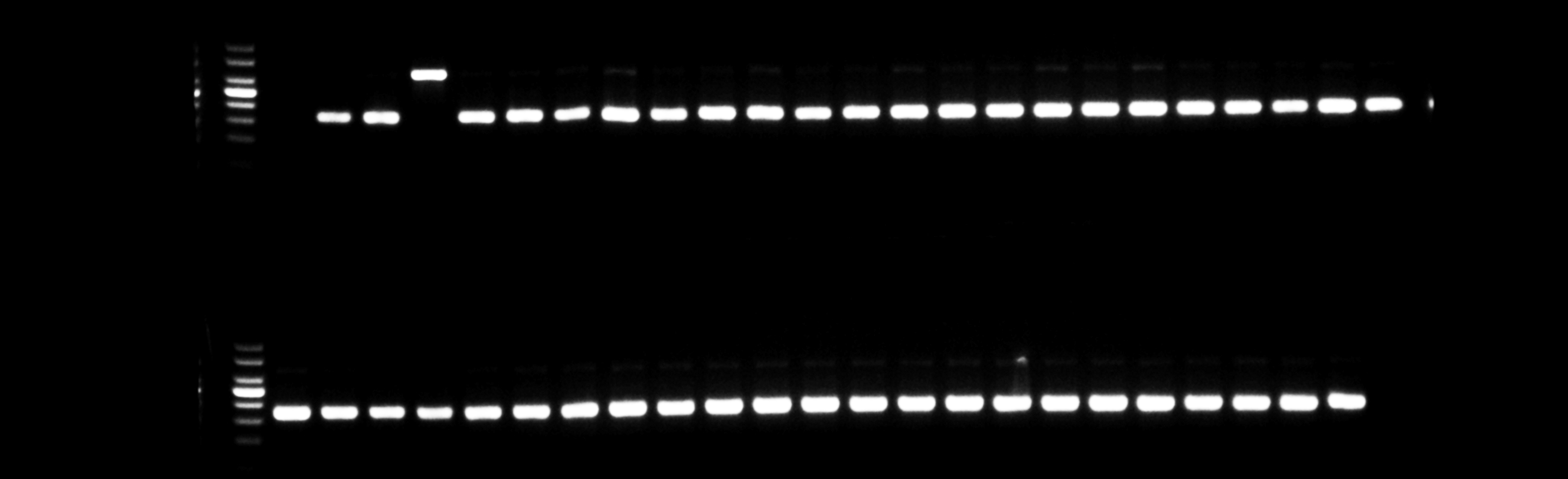

Supplement: Supplementary file 1 [file DataSheet1.zip › Original gels/Figure 3C.tif]

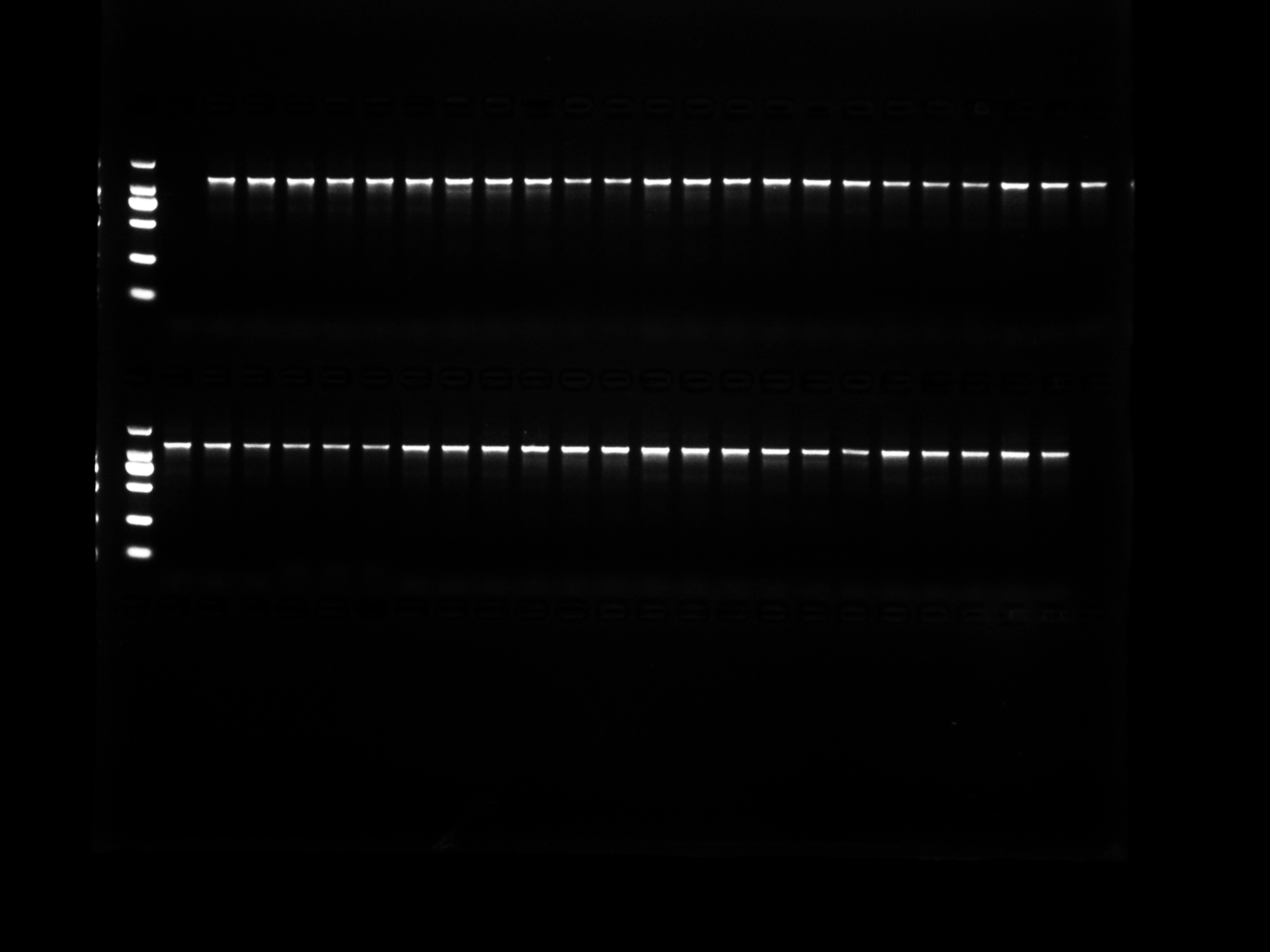

Supplement: Supplementary file 1 [file DataSheet1.zip › Original gels/Figure 3D.tif]

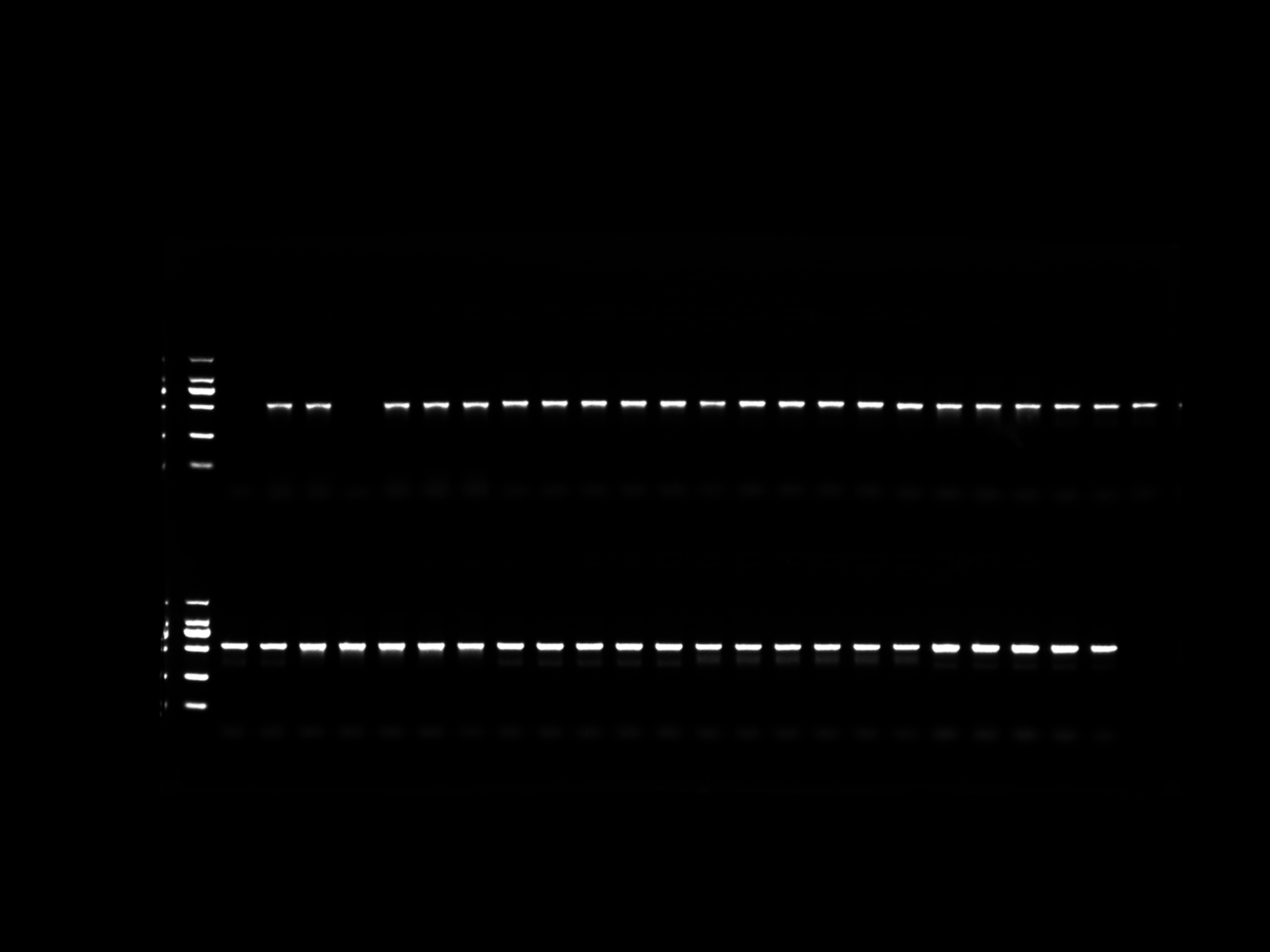

Supplement: Supplementary file 1 [file DataSheet1.zip › Original gels/Figure 3E.tif]
