# Supplementary material for: Genetic characteristics of the diploid offsprings in potato Cooperation 88 induced by diploid donor IVP101
Source: Front Plant Sci. 2024 Nov 8;15:1486549. doi: 10.3389/fpls.2024.1486549 (PMC11582670; doi:10.3389/fpls.2024.1486549)
Supplement: Supplementary file 2 [file Table1.docx]

Supplementary Tables

**Supplementary Table 1.** Source of chloroplast genome data for 12 *Solanum* species.

| **Species** | **Variety name** | **Origin** | **Data sources** | **Accession No.** | **References** |
| --- | --- | --- | --- | --- | --- |
| *S. lycopersicum* | IPA-6 | Golm, Germany | GenBank | AC_000188.1 | Kahlau et al., 2006 |
| *S. melongena* | — | Kunming, China | GenBank | NC_030207.1 | — |
| *S. tuberosum* | Desiree | Portici, Italy | GenBank | NC_008096.2 | Chung et al., 2006 |
| *S. verrucosum* | PI 195170 | Madison, USA | GenBank | NC_041632.1 | — |
| *S. pimpinellifolium* | TS-415 | Fort Collins, USA | GenBank | NC_026882.1 | Wu, 2016 |
| *S. pennellii* | — | Daegu, Korea | GenBank | NC_035742.1 | — |
| [*S. americanum*](https://www.ncbi.nlm.nih.gov/nuccore/NC_062693.1) | — | Helsinki, Finland | GenBank | NC_062693.1 | — |
| [*S. commersonii*](https://www.ncbi.nlm.nih.gov/datasets/taxonomy/4109) | Lz3.2 | Seoul, Korea | GenBank | NC_028069.2 | — |
| [*S. habrochaites*](https://www.ncbi.nlm.nih.gov/datasets/taxonomy/62890) | TS-407 | Fort Collins, USA | GenBank | NC_026879.1 | Wu, 2016 |
| [*S. pinnatisectum*](https://www.ncbi.nlm.nih.gov/datasets/taxonomy/50273) | PI 253214 | Madison, USA | GenBank | NC_041626.1 | — |
| [*S. stenotomum*](https://www.ncbi.nlm.nih.gov/datasets/taxonomy/172797) | PI 320364 | Madison, USA | GenBank | NC_041607.1 | — |
| [*S. chilense*](https://www.ncbi.nlm.nih.gov/datasets/taxonomy/4083) | TS-408 | Fort Collins, USA | GenBank | NC_026877.1 | Wu, 2016 |

**Supplementary Table 2.** InDel sites of IVP101 chloroplast genome.

| **Start** | **End** | **Ref(C88)** | **Start** | **End** | **Alt(IVP101)** | **Gene** |
| --- | --- | --- | --- | --- | --- | --- |
| 449 | 449 | G | 449 | 451 | GAA |  |
| 2098 | 2098 | C | 2100 | 2111 | CTTCGTCATTTA | *trn*K-UUU |
| 3798 | 3799 | CT | 3811 | 3811 | C | *trn*K-UUU |
| 4622 | 4622 | G | 4634 | 4635 | GA |  |
| 6398 | 6425 | CATTATAATATTAATTAATATTAATTAA | 6411 | 6411 | C |  |
| 6692 | 6692 | T | 6678 | 6679 | TA |  |
| 6957 | 6958 | CT | 6944 | 6944 | C |  |
| 7153 | 7153 | G | 7139 | 7140 | GA |  |
| 7202 | 7250 | ATTCCAGCTAACATTTCGATTTTAAACAGAACATTGTTAAAAAAGCAAT | 7189 | 7189 | A |  |
| 8201 | 8201 | C | 8140 | 8142 | CTT |  |
| 8449 | 8450 | GA | 8390 | 8390 | G |  |
| 9778 | 9782 | CTTTT | 9718 | 9718 | C | *trn*G-UCC |
| 9977 | 9977 | T | 9913 | 9922 | TTTATTCTAA |  |
| 10036 | 10066 | TATTAGTACATCATTGAATATACAATTCAAA | 9981 | 9981 | T |  |
| 14417 | 14417 | T | 14332 | 14333 | TA |  |
| 14668 | 14668 | C | 14584 | 14586 | CTT |  |
| 16751 | 16751 | T | 16669 | 16670 | TC |  |
| 16787 | 16787 | A | 16706 | 16709 | AAAG |  |
| 27777 | 27777 | T | 27699 | 27700 | TC |  |
| 29181 | 29187 | TCATATA | 29104 | 29104 | T |  |
| 32310 | 32310 | A | 32227 | 32232 | ATTTTT |  |
| 33333 | 33333 | C | 33255 | 33259 | CTATT |  |
| 36313 | 36315 | CTT | 36239 | 36239 | C |  |
| 43251 | 43258 | ATAATTTT | 43175 | 43175 | A |  |
| 43341 | 43341 | A | 43258 | 43269 | AGTGAAATAGAA |  |
| 47737 | 47738 | GA | 47665 | 47665 | G |  |
| 53089 | 53090 | AT | 53016 | 53016 | A | *trn*V-UAC |
| 53488 | 53488 | A | 53414 | 53415 | AC | *trn*V-UAC |
| 56578 | 56579 | CA | 56505 | 56505 | C |  |
| 58673 | 58673 | A | 58599 | 58606 | AGATTTTC |  |
| 60544 | 60544 | A | 60477 | 60478 | AT |  |
| 60712 | 60720 | CCTTTATCA | 60646 | 60646 | C |  |
| 62571 | 62571 | T | 62497 | 62506 | TTTTTTTTAG |  |
| 68117 | 68118 | CA | 68052 | 68052 | C |  |
| 70414 | 70430 | AGAATCTAGATTTGAGT | 70348 | 70348 | A |  |
| 71635 | 71636 | GT | 71553 | 71553 | G |  |
| 72581 | 72582 | TC | 72498 | 72498 | T | *rps*12 |
| 72748 | 72748 | A | 72664 | 72671 | AAAAAAAC | *rps*12 |
| 74068 | 74068 | A | 73991 | 73992 | AT | *rps*12 |
| 76120 | 76121 | TA | 76044 | 76044 | T | *rps*12 |
| 82978 | 82978 | G | 82901 | 82902 | GA | *rps*12 |
| 84586 | 84586 | C | 84510 | 84511 | CT | *rps*12 |
| 114411 | 114411 | A | 114336 | 114338 | AAC | *rps*12 |
| 114817 | 114817 | C | 114744 | 114752 | CTTCAATTA | *rps*12 |
| 115438 | 115446 | GAGTATAGA | 115373 | 115373 | G | *rps*12 |
| 115509 | 115518 | CAGGTTAAAT | 115436 | 115436 | C | *rps*12 |
| 120467 | 120467 | T | 120385 | 120395 | TACTATTATAA | *rps*12 |

**Supplementary Table 3.** SNP site of IVP101 chloroplast genome.

| **Pos** | **Ref(C88)** | **Pos** | **Alt(IVP101)** | **Pos** | **Ref(C88)** | **Pos** | **Alt(IVP101)** |
| --- | --- | --- | --- | --- | --- | --- | --- |
| 743 | A | 745 | G | 68520 | T | 68454 | C |
| 1810 | G | 1812 | T | 68593 | C | 68527 | A |
| 2660 | T | 2673 | C | 68594 | T | 68528 | A |
| 3085 | C | 3098 | A | 68595 | T | 68529 | A |
| 3276 | G | 3289 | A | 68596 | T | 68530 | G |
| 3495 | C | 3508 | A | 68719 | A | 68653 | G |
| 4108 | C | 4120 | A | 69584 | G | 69518 | T |
| 4130 | C | 4142 | T | 70804 | G | 70722 | T |
| 4429 | T | 4441 | G | 71900 | A | 71817 | G |
| 4435 | C | 4447 | T | 72377 | G | 72294 | T |
| 4638 | G | 4651 | T | 72516 | C | 72433 | A |
| 4878 | T | 4891 | C | 73368 | G | 73291 | T |
| 6720 | G | 6707 | A | 77263 | A | 77186 | C |
| 7067 | G | 7053 | T | 78365 | A | 78288 | C |
| 8020 | C | 7959 | T | 78769 | T | 78692 | A |
| 8298 | T | 8239 | C | 78770 | T | 78693 | A |
| 9154 | C | 9094 | T | 81227 | A | 81150 | G |
| 9349 | A | 9289 | G | 81861 | A | 81784 | G |
| 10236 | G | 10151 | T | 82153 | G | 82076 | A |
| 10313 | T | 10228 | G | 82775 | C | 82698 | T |
| 10472 | C | 10387 | T | 84166 | T | 84090 | C |
| 11609 | G | 11524 | A | 84363 | G | 84287 | T |
| 12622 | A | 12537 | C | 84638 | G | 84563 | T |
| 17987 | C | 17909 | A | 84642 | G | 84567 | T |
| 20154 | G | 20076 | C | 84665 | G | 84590 | A |
| 21345 | G | 21267 | A | 84983 | A | 84908 | T |
| 22619 | T | 22541 | C | 85151 | G | 85076 | T |
| 23068 | G | 22990 | C | 85423 | A | 85348 | G |
| 23549 | A | 23471 | G | 85552 | C | 85477 | A |
| 23612 | G | 23534 | T | 85752 | C | 85677 | T |
| 25236 | C | 25158 | A | 90076 | C | 90001 | A |
| 25544 | C | 25466 | T | 95116 | C | 95041 | T |
| 26085 | G | 26007 | A | 107266 | T | 107191 | C |
| 27212 | A | 27134 | C | 111556 | G | 111481 | T |
| 27306 | T | 27228 | A | 111786 | G | 111711 | T |
| 27441 | A | 27363 | C | 111872 | T | 111797 | G |
| 27768 | G | 27690 | T | 113310 | A | 113235 | G |
| 28035 | C | 27958 | A | 113828 | A | 113753 | G |
| 28389 | G | 28312 | T | 114227 | G | 114152 | T |
| 28787 | T | 28710 | G | 114286 | C | 114211 | A |
| 29445 | G | 29362 | A | 114581 | A | 114508 | G |
| 31404 | G | 31321 | T | 114797 | G | 114724 | T |
| 33009 | C | 32931 | G | 115299 | G | 115234 | A |
| 33417 | C | 33343 | A | 115840 | T | 115758 | G |
| 33658 | G | 33584 | A | 116475 | C | 116393 | A |
| 36110 | T | 36036 | C | 118027 | G | 117945 | T |
| 42599 | A | 42523 | G | 119539 | C | 119457 | A |
| 43034 | C | 42958 | T | 120031 | T | 119949 | C |
| 44722 | G | 44650 | T | 120105 | T | 120023 | C |
| 47284 | C | 47212 | A | 120296 | A | 120214 | C |
| 47576 | G | 47504 | A | 121560 | G | 121488 | T |
| 47989 | T | 47916 | A | 121855 | C | 121783 | T |
| 48383 | C | 48310 | A | 121905 | C | 121833 | A |
| 48438 | C | 48365 | G | 122411 | C | 122339 | T |
| 49212 | C | 49139 | A | 122441 | T | 122369 | C |
| 51839 | T | 51766 | G | 122612 | A | 122540 | C |
| 52123 | T | 52050 | C | 122662 | T | 122590 | C |
| 55699 | C | 55626 | T | 122804 | T | 122732 | C |
| 55719 | G | 55646 | T | 123168 | C | 123096 | T |
|  |  |  |  |  |  |  |  |
| **Supplementary Table 2.** *Continued.* | | | | | | | |
| **Pos** | **Ref(C88)** | **Pos** | **Alt(IVP101)** | **Pos** | **Ref(C88)** | **Pos** | **Alt(IVP101)** |
| 56253 | T | 56180 | G | 123380 | C | 123308 | T |
| 56287 | C | 56214 | T | 123817 | T | 123745 | C |
| 56784 | C | 56710 | T | 124734 | G | 124662 | C |
| 57175 | C | 57101 | T | 124789 | G | 124717 | C |
| 57250 | G | 57176 | A | 124992 | T | 124920 | C |
| 57251 | T | 57177 | C | 125072 | A | 125000 | C |
| 57514 | G | 57440 | A | 125073 | G | 125001 | T |
| 57610 | A | 57536 | G | 125576 | T | 125504 | C |
| 57611 | C | 57537 | T | 126611 | G | 126539 | T |
| 57753 | G | 57679 | C | 127104 | T | 127032 | A |
| 58142 | G | 58068 | A | 127133 | G | 127061 | T |
| 58171 | G | 58097 | T | 127322 | T | 127250 | C |
| 58172 | C | 58098 | G | 127747 | T | 127675 | A |
| 58612 | G | 58538 | T | 128004 | C | 127932 | A |
| 60809 | C | 60735 | A | 128072 | G | 128000 | T |
| 61140 | T | 61066 | A | 128178 | T | 128106 | G |
| 62474 | G | 62400 | T | 128391 | G | 128319 | T |
| 62629 | G | 62564 | T | 129300 | A | 129228 | C |
| 62707 | C | 62642 | T | 129353 | C | 129281 | T |
| 62793 | G | 62728 | T | 129507 | T | 129435 | C |
| 62874 | A | 62809 | G | 129634 | T | 129562 | G |
| 62927 | A | 62862 | G | 129864 | T | 129792 | C |
| 63823 | G | 63758 | A | 130014 | C | 129942 | A |
| 67471 | C | 67406 | A | 134304 | A | 134232 | G |
| 67517 | C | 67452 | A | 146454 | G | 146382 | A |
| 68294 | G | 68228 | A | 151494 | G | 151422 | T |

**Supplementary Table 4.** Nucleotide diversity of chloroplast genomes of 14 *Solanum* species (Pi > 0.01500).

| **Window** | **Midpoint** | **Pi** | **S** | **Gene(Based on the midpoint pos)** |
| --- | --- | --- | --- | --- |
| 64937-65350 | 65136 | 0.03937 | 56 | *cem*A |
| 64737-65136 | 64936 | 0.03547 | 50 | *cem*A |
| 57401-57959 | 57696 | 0.03445 | 71 | *atp*B-*rbc*L |
| 57197-57696 | 57400 | 0.03047 | 60 | *atp*B-*rbc*L |
| 73477-73876 | 73676 | 0.03033 | 40 | *clp*P(exon3) |
| 130582-130981 | 130781 | 0.02898 | 50 | *ycf*1 |
| 131385-131784 | 131584 | 0.02821 | 46 | *ycf*1 |
| 129573-129981 | 129775 | 0.02739 | 38 | *ycf*1 |
| 116072-116505 | 116288 | 0.0267 | 44 | *ndh*F-*rpl*32 |
| 130782-131184 | 130981 | 0.02648 | 48 | *rpl*32 |
| 115865-116288 | 116071 | 0.02637 | 41 | *ndh*F-*rpl*32 |
| 129776-130181 | 129981 | 0.02629 | 42 | *ycf*1 |
| 130982-131384 | 131184 | 0.02566 | 47 | *ycf*1 |
| 131185-131584 | 131384 | 0.02536 | 45 | *ycf*1 |
| 116706-117151 | 116936 | 0.02519 | 37 | *rpl*32-*trn*L |
| 128155-128566 | 128354 | 0.02516 | 39 | *ycf*1 |
| 4547-5097 | 4893 | 0.02500 | 33 | *trn*K(exon1)-*rps*16(exon2) |
| 116506-116936 | 116705 | 0.02478 | 35 | *rpl*32 |
| 74524-74924 | 74723 | 0.02473 | 44 | *clp*P(exon2) |
| 129982-130381 | 130181 | 0.02453 | 43 | *ycf*1 |
| 117152-117633 | 117397 | 0.02393 | 37 | *rpl*32-*trn*L |
| 1-511 | 289 | 0.02390 | 38 | *trn*H-*psb*A |
| 130382-130781 | 130581 | 0.02360 | 36 | *ycf*1 |
| 290-718 | 511 | 0.02192 | 35 | *trn*H-*psb*A |
| 128355-128766 | 128566 | 0.02181 | 37 | *ycf*1 |
| 6693-7314 | 7101 | 0.02162 | 39 | *rps*16(exon1)-*trn*Q |
| 129373-129775 | 129572 | 0.02104 | 34 | *ycf*1 |
| 130182-130581 | 130381 | 0.02060 | 30 | *ycf*1 |
| 131585-131984 | 131784 | 0.02003 | 32 | *ycf*1 |
| 116289-116705 | 116505 | 0.01995 | 33 | *ndh*F-*rpl*32 |
| 128967-129372 | 129172 | 0.01989 | 35 | *ycf*1 |
| 127141-127554 | 127340 | 0.01973 | 31 | *rps*15-*ycf*1 |
| 116937-117397 | 117151 | 0.01956 | 34 | *rpl*32-*trn*L |
| 118634-119051 | 118833 | 0.01931 | 28 | *ccs*A |
| 59565-60006 | 59769 | 0.01907 | 28 | *rbc*L-*acc*D |
| 126941-127340 | 127140 | 0.01898 | 27 | *rps*15-*ycf*1 |
| 50377-50891 | 50666 | 0.01893 | 32 | *trn*F-*ndh*J |
| 128767-129172 | 128966 | 0.01882 | 34 | *ycf*1 |
| 115665-116071 | 115864 | 0.01832 | 27 | *ndh*F-*rpl*32 |
| 30502-30912 | 30701 | 0.01830 | 23 | *pet*N-*psb*M |
| 57697-58164 | 57959 | 0.01827 | 30 | *atp*B-*rbc*L |
| 50667-51161 | 50891 | 0.01824 | 26 | *trn*F-*ndh*J |
| 4343-4893 | 4546 | 0.01813 | 27 | *trn*K(exon1)-*rps*16(exon2) |
| 74314-74723 | 74523 | 0.01810 | 32 | *clp*P(exon2) |
| 129173-129572 | 129372 | 0.01805 | 31 | *ycf*1 |
| 6315-7101 | 6692 | 0.01802 | 38 | *rps*16(exon1)-*trn*Q |
| 62048-62461 | 62255 | 0.01797 | 27 | *acc*D-*psa*I |
| 114065-114464 | 114264 | 0.01775 | 33 | *ndh*F |
| 28415-28833 | 28623 | 0.01753 | 24 | *rpo*B-*trn*C |
| 118834-119251 | 119051 | 0.01736 | 30 | *ccs*A-*ndh*D |
| 75564-75977 | 75766 | 0.01734 | 35 | *clp*P(exon1)-*psb*B |
| 59365-59769 | 59564 | 0.01709 | 20 | *rbc*L |
| 2731-3130 | 2930 | 0.01703 | 35 | *mat*K |
| 74724-75131 | 74924 | 0.01690 | 31 | *clp*P(exon2)-*clp*P(exon1) |
| 63894-64336 | 64125 | 0.01670 | 25 | *ycf*4-*cem*A |
| 61844-62255 | 62047 | 0.01657 | 27 | *acc*D-*psa*I |
| 127955-128354 | 128154 | 0.01615 | 29 | *ycf*1 |
| 3340-3739 | 3539 | 0.01599 | 24 | *mat*K |
| 75350-75766 | 75563 | 0.01585 | 34 | *clp*P(exon2)-*clp*P(exon1) |
|  |  |  |  |  |
| **Supplementary Table 3.** *Continued.* | | | | |
| **Window** | **Midpoint** | **Pi** | **S** | **Gene(Based on the midpoint pos)** |
| 123881-124316 | 124080 | 0.01585 | 29 | *ndh*A(exon2)-*ndh*A(exon1) |
| 9034-9464 | 9262 | 0.01571 | 28 | *trn*S-*trn*R |
| 86134-86569 | 86368 | 0.01571 | 25 | *rpl*16(exon1)-*rps*3 |
| 113453-113864 | 113664 | 0.01569 | 21 | *ndh*F |
| 124081-124523 | 124316 | 0.01533 | 26 | *ndh*A(exon2)-*ndh*A(exon1) |
| 66776-67235 | 66987 | 0.01525 | 34 | *pet*A-*psb*J |
